# The Presence of Nuclear Cactus in the Early *Drosophila* Embryo May Extend the Dynamic Range of the Dorsal Gradient

**DOI:** 10.1371/journal.pcbi.1004159

**Published:** 2015-04-16

**Authors:** Michael D. O’Connell, Gregory T. Reeves

**Affiliations:** 1 North Carolina State University Department of Chemical and Biomolecular Engineering, Raleigh, North Carolina, United States of America; University of Oxford, UNITED KINGDOM

## Abstract

In a developing embryo, the spatial distribution of a signaling molecule, or a morphogen gradient, has been hypothesized to carry positional information to pattern tissues. Recent measurements of morphogen distribution have allowed us to subject this hypothesis to rigorous physical testing. In the early Drosophila embryo, measurements of the morphogen Dorsal, which is a transcription factor responsible for initiating the earliest zygotic patterns along the dorsal-ventral axis, have revealed a gradient that is too narrow to pattern the entire axis. In this study, we use a mathematical model of Dorsal dynamics, fit to experimental data, to determine the ability of the Dorsal gradient to regulate gene expression across the entire dorsal-ventral axis. We found that two assumptions are required for the model to match experimental data in both Dorsal distribution and gene expression patterns. First, we assume that Cactus, an inhibitor that binds to Dorsal and prevents it from entering the nuclei, must itself be present in the nuclei. And second, we assume that fluorescence measurements of Dorsal reflect both free Dorsal and Cactus-bound Dorsal. Our model explains the dynamic behavior of the Dorsal gradient at lateral and dorsal positions of the embryo, the ability of Dorsal to regulate gene expression across the entire dorsal-ventral axis, and the robustness of gene expression to stochastic effects. Our results have a general implication for interpreting fluorescence-based measurements of signaling molecules.

## Author Summary

In the early stages of development, an embryo must establish the beginnings of a body plan in order to grow organs and limbs in the right places. Across the fruit fly embryo, proteins known as morphogens cause chain reactions that eventually lead to the growth of many different body parts. One specific morphogen, known as Dorsal, is distributed in a gradient across the dorsal-ventral (DV) axis of the embryo. The Dorsal gradient has recently been measured in live embryos using a fluorescently-tagged version of Dorsal, but the gradient measured by fluorescence microscopy seems to be too narrow to pattern all of the DV axis. Using a mathematical model of the *Drosophila* embryo, we have proposed a solution to this outstanding problem: namely that Cactus, the inhibitor to Dorsal, is present with Dorsal in nuclei across the embryo, which creates a disparity between the gradient measured by fluorescence and the gradient measured by gene expression. By using distinct model equations for active and inactive pools of Dorsal, we were able to recreate the dynamics of the Dorsal gradient and the expression patterns of its target genes with a high level of accuracy, showing that mathematical models may be critical for properly interpreting fluorescence data.

## Introduction

Tissues in a developing animal are often patterned by long-range signaling factors called morphogens. In the early (1–3 hr old) *Drosophila* embryo, the transcription factor Dorsal (dl; FlyBase ID: FBgn0260632) acts as a morphogen to pattern the embryo’s dorsal-ventral (DV) axis ([1, 2]; reviewed in [3, 4]). dl, a homologue of mammalian NF-*κ*B, is expressed ubiquitously throughout the syncitial blastoderm, and is sequestered to the cytoplasm by its association with the inhibitor Cactus (Cact; FlyBase: FBgn0000250), a homologue of mammalian I*κ*B [5]. Signaling through Toll receptors on the ventral side of the embryo causes the dissociation of the dl/Cact complex, and free dl accumulates in the ventral nuclei [5–7] to create a spatial gradient that causes differential gene expression based on multiple gene expression thresholds.

dl influences the expression of 50+ genes, which have been categorized into Types I, II, and III+/- based on their domains of expression (reviewed in [3, 8]). Types I, II and III+ (such as *sna*, *vnd* and *sog*; FlyBase: FBgn0003448, FBgn0261930, FBgn0003463) are activated by high, moderate, and low levels of nuclear dl, respectively, and thus form boundaries at roughly 20%, 33%, and 50% DV position (with 0% DV being the ventral midline). Type III- genes (such as *dpp* and *zen*; FlyBase: FBgn0000490, FBgn0004053) are repressed by dl, and thus are expressed in nuclei on the dorsal half of the embryo where there is little or no dl [9, 10].

In recent years, detailed measurements of the dl gradient have been performed, potentially allowing us to address the question of how the spatial information carried by the dl gradient results in gene expression [10–12]. Quantitative measurements in fixed embryos revealed a dl gradient that becomes flat by ∼ 40% DV position, calling into question how such a short-ranged gradient could pattern the Type III genes [12]. However, these results were in contrast to classical enhancer trap studies that suggested that dl directly sets the border of the Type III- gene *zen* [13]. One proposed explanation to rectify this difference is the dl gradient width may be dynamic, as in the case of *Tribolium* [14].

Indeed, pioneering work using a GFP-tagged dl revealed the dynamic nature of the dl gradient, in which dl levels slowly build in ventral nuclei during interphase, then sharply drop during mitosis when the nuclei divide, only to begin the pattern again at the start of the next interphase [11]. This “saw tooth” observation was later confirmed by both detailed measurements in fixed embryos [12], as well as modeling work [15]. In contrast, dl levels in the dorsal-most nuclei were found to slowly decrease during interphase, and recover back at the start of the next interphase [10]. However, while the nuclear concentration of dl was found to be highly dynamic all along the DV axis, the seemingly narrow width of the dl gradient was measured to be constant [10, 12].

These observations left open the question of how a narrow-width dl gradient can specify gene expression domains far beyond its apparent spatial range [10, 12, 16]. To determine the answers to these questions, we analyze a model of dl/Cact dynamics. Our model is based on previously-published work [15], and makes two distinctive assumptions: first, that Cact can be present in the nuclei to regulate dl; and second, that fluorescence measurements (from either dl immunofluorescence experiments or from fluorescent protein-tagged dl) comprise both free and Cact-bound dl. Under these two assumptions, our model, when fit to the spatiotemporal measurements in live embryos [10], can explain fine details of the dynamics of the dl gradient. Using this result as a starting point, we simulated dl-dependent gene expression, taking into account the fact that only the free (and not Cact-bound) nuclear dl can regulate gene expression. We found that nuclei can accurately interpret their position along the DV axis even in regions of the embryo where stochasticity causes high levels of gradient read-out error. Thus, our model explains both the dynamics of the dl gradient as well as its spatial range. Our results have implications on how fluorescence measurements should be interpreted, in particular when there is a binding partner that modulates the activity of a signaling molecule.

## Methods

### dl/Cact model formulation

**Fig 1:**
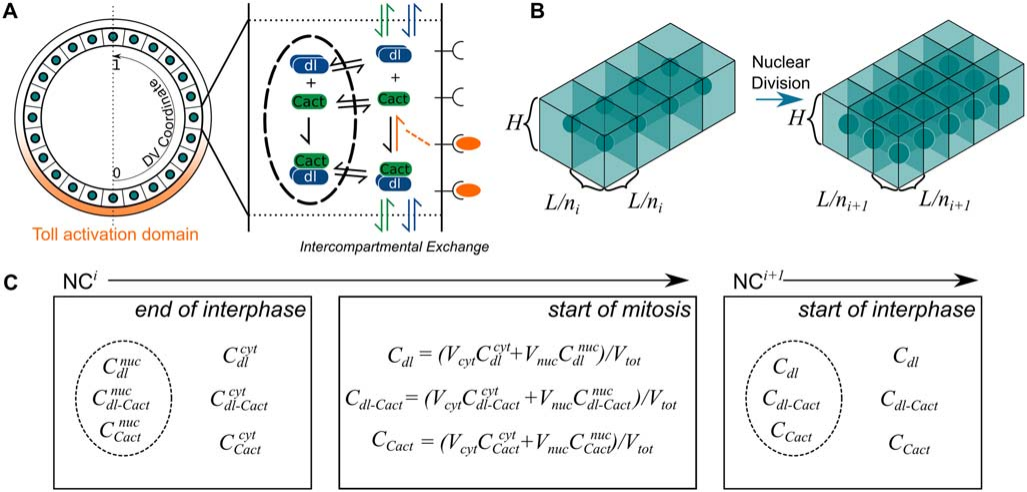
Description of geometry. (a) The model consists of a linear array of compartments, each containing a single nucleus, representing one-half of the DV axis. All three species can enter and exit the nuclei and diffuse across the compartmental wall. The Toll signal is represented by a Gaussian curve along the ventral half of the embryo. (b) At the beginning of each nuclear cycle, the number of nuclear compartments increases by $\sqrt{2}$, to the nearest integer. The height of the compartments remains constant, while the length/width is calculated by the length of the compartmental array divided by *n*_*i*_, the number of nuclei in nuclear cycle *i*. (c) At the end of interphase, the nuclear concentration of each protein is distinct from its cytoplasmic concentration. At the start of mitosis, the nuclear and cytoplasmic protein concentrations from the end of interphase are mixed. At the start of the next interphase, the concentration profile of a nucleus is initially the same as the surrounding cytoplasm.

We begin our model formulation from a previously published model [15], and make some adjustments for consistency with recently-acquired data [10]. Here we sketch the essentials for understanding the model. (For full details, see S1 Text.) To simulate the dynamics of dl and Cact during NC10-NC14, a cross section of the embryo was modeled as a linear array of rectangular prism-shaped compartments that each contain a single nucleus (Fig. 1a,b). Each compartment and each nucleus are well mixed, with slow exchange between neighboring compartments [11, 17]. Because the embryo is approximately symmetric about the DV axis, only one half of the axis is simulated and no-exchange boundary conditions are assumed at both the ventral and dorsal midlines. The number of compartments, as well as their dimensions, depends on the number of nuclei, which increases (to the nearest integer) by a factor of $\sqrt{2}$ at the start of each interphase (Fig. 1b). The length and width of each compartment is calculated as the length of the simulated region, *L*, divided by the number of nuclei in interphase *i*, *n*_*i*_; the height, *H*, remains constant.

The simulation begins at the onset of NC10 interphase with the initial conditions for each molecular species uniform in space. The normalized concentration of nuclear/cytoplasmic Cact is at steady state; the concentration of dl/Cact complex is unity; and the concentration of free dl is zero.

**Fig 2:**
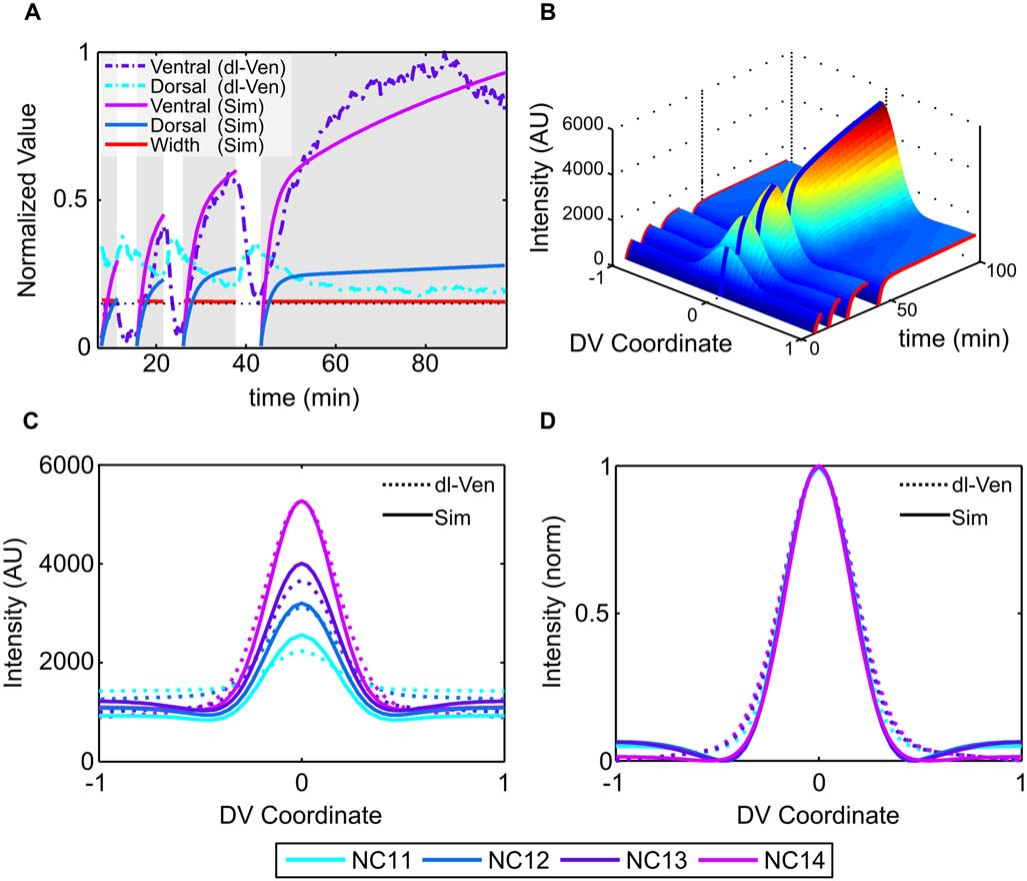
Model results without nuclear dl/Cact. (a) Simulations of the model assuming nuclei begin interphase empty and that only free cytoplasmic dl is imported into the nucleus. (b) 3D surface plot of the best-fit parameter set. (c) Snapshots of the end of each nuclear cycle according to this model. (d) Same as in (c), normalized.

The following description is for the full model that includes dl/Cact complex and Cact in the nuclei. (For a description of the initial model used—depicted in Fig. 2—see S1 Text.) During interphase, the nuclear and cytoplasmic concentrations are governed by the parameters associated with nuclear import/export, intercompartmental exchange, production/degradation of Cact, and binding/unbinding of dl/Cact complex. At the start of mitosis the nuclei break down and the contents of each nucleus are mixed with that of the surrounding cytoplasmic compartments (Fig. 1c, center panel). The equations for the mitosis phase are subsequently solved for the appropriate duration, and the next interphase begins with nuclei and cytoplasmic compartments having the same concentration (Fig. 1c, right panel). This allows for the presence of all three molecular species (dl, Cact and dl/Cact complex) to exist in the nuclei at the start of interphase.

The surface area and volume of each nucleus, while dynamic over the course of the entire simulation, are considered static for the duration of each NC for simplicity. Nuclear dimensions are calculated using published measurements ([18]; for full description see S1 Text).

Interphase and mitosis dynamics are represented by six and three non-dimensionalized differential equations, respectively, consisting of up to 15 free parameters (see Box 1; see S1 Table and S2 Table for dimensional analysis). During interphase, each of the three molecular species is represented by two equations, one for the nuclear concentration and one for the cytoplasmic concentration. During mitosis the nuclei are undefined, so only the equations representing cytoplasmic concentrations are solved. When appropriate, the import/export rates (*ζ*_*i*_, *ξ*_*i*_), intercompartmental exchange rates (*λ*_*i*_), dl/Cact binding (*γ*) and dissociation (*β*_0_) rates, and degradation of Cact (*α*) are all represented by free parameters. The import and export rates of each species are approximated as a first-order process, and exchange is formulated as mass transport flux between neighboring compartments. In addition, the Toll-mediated dissociation rate of dl/Cact complex is modeled as a Gaussian curve centered on the ventral midline ($\beta e^{-\frac{1}{2}{\left(\frac{x}{\phi }\right)}^{2}}$), which represents the distribution of active Toll receptors. The intensity and range of Toll-mediated dissociation is specified by the amplitude and width parameters (*β*, *ϕ*), respectively.

**Box 1**

$$\begin{array}{ccc} \frac{d\left({\tilde{V}}_{n}U_{nuc}^{h}\right)}{dt} & = & {\tilde{A}}_{n}\left(\zeta_{dl}U_{cyt}^{h}-\xi_{dl}U_{nuc}^{h}\right)-{\tilde{V}}_{n}\left(\gamma U_{nuc}^{h}Z_{nuc}-\beta_{0}W_{nuc}^{h}\right) \end{array}$$ (1)

$$\begin{array}{ccc} \frac{d\left({\tilde{V}}_{C}U_{cyt}^{h}\right)}{dt} & = & {\tilde{A}}_{m}\lambda_{dl}\left(U_{cyt}^{h-1}-2U_{cyt}^{h}+C_{cyt}^{h+1}\right)+{\tilde{V}}_{C}\left(\hat{\beta }W_{cyt}^{h}-\gamma U_{cyt}^{h}Z_{cyt}^{h}\right)\\ & & -{\tilde{A}}_{n}\left(\zeta_{dl}U_{cyt}^{h}-\xi_{dl}U_{nuc}^{h}\right) \end{array}$$ (2)

$$\begin{array}{ccc} \frac{d\left({\tilde{V}}_{n}W_{nuc}^{h}\right)}{dt} & = & {\tilde{A}}_{n}\left(\zeta_{dc}W_{cyt}^{h}-\xi_{dc}W_{nuc}^{h}\right)+{\tilde{V}}_{n}\left(\gamma U_{nuc}^{h}Z_{nuc}^{h}-\beta_{0}W_{nuc}^{h}\right) \end{array}$$ (3)

$$\begin{array}{ccc} \frac{d\left({\tilde{V}}_{C}W_{cyt}^{h}\right)}{dt} & = & {\tilde{A}}_{m}\lambda_{dc}\left(W_{cyt}^{h-1}-2W_{cyt}^{h}+W_{cyt}^{h+1}\right)-{\tilde{V}}_{C}\left(\hat{\beta }W_{cyt}^{h}-\gamma U_{cyt}^{h}Z_{cyt}^{h}\right)\\ & & -{\tilde{A}}_{n}\left(\zeta_{dc}W_{cyt}-\xi_{dc}W_{nuc}\right) \end{array}$$ (4)

$$\begin{array}{ccc} \frac{d\left({\tilde{V}}_{n}Z_{nuc}^{h}\right)}{dt} & = & {\tilde{A}}_{n}\left(\zeta_{cact}Z_{cyt}^{h}-\xi_{cact}Z_{nuc}^{h}\right)-{\tilde{V}}_{n}\psi \left(\gamma U_{nuc}^{h}Z_{nuc}^{h}-\beta_{0}W_{nuc}^{h}\right) \end{array}$$ (5)

$$\begin{array}{ccc} \frac{d\left({\tilde{V}}_{C}Z_{cyt}^{h}\right)}{dt} & = & {\tilde{A}}_{m}\lambda_{Cact}\left(Z_{cyt}^{h-1}-2Z_{cyt}^{h}+Z_{cyt}^{h+1}\right)+{\tilde{V}}_{C}\left(\psi \hat{\beta }W_{cyt}^{h}-\psi \gamma U_{cyt}^{h}Z_{cyt}^{h}\right.\\ & & \left.-\alpha Z_{cyt}^{h}\right)-{\tilde{A}}_{n}\left(\zeta_{cact}Z_{cyt}^{h}-\xi_{cact}Z_{nuc}^{h}\right)+1 \end{array}$$ (6)

where $\hat{\beta }=\beta exp(-\frac{1}{2}{\left(\frac{z}{\phi^{\prime }}\right)}^{2})+\beta_{0}$ and

$$\begin{array}{c} \zeta_{s}=\frac{A_{n}^{14}k_{in,s}}{V_{n}^{14}}\bar{T}\;\xi_{s}=\frac{A_{n}^{14}k_{out,s}}{V_{n}^{14}}\bar{T}\;\lambda_{s}=\frac{\Gamma_{s}A_{n}^{14}}{V_{n}^{14}}\bar{T}\;\gamma =k_{b}C_{cact}^{0}\bar{T}\;\alpha =k_{Deg}\bar{T}\;\beta =k_{D}^{max}\bar{T}\;\beta_{0}=k_{D0}\bar{T} \end{array}$$

$$\begin{array}{c} U_{nuc}=\frac{C_{dl,n}}{C_{dl}^{0}}\;U_{cyt}=\frac{C_{dl,c}}{C_{dl}^{0}}\;W_{nuc}=\frac{C_{dc,n}}{C_{dl}^{0}}\;W_{cyt}=\frac{C_{dc,c}}{C_{dl}^{0}}\;Z_{nuc}=\frac{C_{Cact,n}}{C_{Cact}^{0}}\;Z_{cyt}=\frac{C_{Cact,c}}{C_{Cact}^{0}} \end{array}$$

$$\begin{array}{c} {\tilde{A}}_{n}=\frac{A_{n}}{A_{n}^{14}}\;{\tilde{A}}_{m}=\frac{A_{m}}{A_{n}^{14}}\;{\tilde{V}}_{n}=\frac{V_{n}}{V_{n}^{14}}\;{\tilde{V}}_{C}=\frac{V_{C}}{V_{n}^{14}}\;t=\frac{T}{\bar{T}}\;\bar{T}=1\;min\;z=\frac{x}{L}\;\phi^{'}=\frac{\phi }{L}\;\psi =\frac{C_{dl}^{0}}{C_{Cact}^{0}} \end{array}$$

for *s* = {*dl*, *n*; *dl*, *c*; *dc*, *n*; *dc*, *c*; *Cact*, *n*; *Cact*, *c*}. For the mitosis phase, we have:

$$\begin{array}{ccc} \frac{d\left({\tilde{V}}_{C}U_{cyt}^{h}\right)}{dt} & = & {\tilde{A}}_{m}\lambda_{dl}\left(U_{cyt}^{h-1}-2U_{cyt}^{h}+C_{cyt}^{h+1}\right)+{\tilde{V}}_{C}\left(\hat{\beta }W_{cyt}^{h}-\gamma U_{cyt}^{h}Z_{cyt}^{h}\right) \end{array}$$ (7)

$$\begin{array}{ccc} \frac{d\left({\tilde{V}}_{C}W_{cyt}^{h}\right)}{dt} & = & {\tilde{A}}_{m}\lambda_{dc}\left(W_{cyt}^{h-1}-2W_{cyt}^{h}+W_{cyt}^{h+1}\right)-{\tilde{V}}_{C}\left(\hat{\beta }W_{cyt}^{h}-\gamma U_{cyt}^{h}Z_{cyt}^{h}\right) \end{array}$$ (8)

$$\begin{array}{ccc} \frac{d\left({\tilde{V}}_{C}Z_{cyt}^{h}\right)}{dt} & = & {\tilde{A}}_{m}\lambda_{Cact}\left(Z_{cyt}^{h-1}-2Z_{cyt}^{h}+Z_{cyt}^{h+1}\right)+{\tilde{V}}_{C}\left(\psi \hat{\beta }W_{cyt}^{h}-\psi \gamma U_{cyt}^{h}Z_{cyt}^{h}-\alpha Z_{cyt}^{h}\right)+1 \end{array}$$ (9)

### Gene expression model

To simulate gene expression, we used a simple linear production-degradation model for each of the four species (*sna, vnd, sog, zen*) in nucleus *h*: $\frac{d}{dt}{\left[mRNA\right]}_{i}^{h}=\frac{1}{\tau_{i}}\left(f_{i}\left(U_{nuc}^{h},{\left[sna\right]}^{h}\right)-{\left[mRNA\right]}_{i}^{h}\right)$, where *f* is the production rate of species *i*, and $U_{nuc}^{h}$ is the scaled concentration of nuclear dl in nucleus *h* [10, 19]. For simplicity, *f* is formulated as a hard-threshold on/off switch for each species (Hill function with Hill exponent of *n*_*H*_ = 100), where production is unity when the concentration of dl is above (for *sna/sog/vnd*) or below (for *zen*) a threshold *θ*_*dl*:*mRNA*_*i*__. In addition, production of *vnd* and *sog* is repressed by *sna* above a threshold *θ*_*sna*:*mRNA*_*i*__. To simulate the error in gradient interpretation in each nucleus due to the stochasticity of the arrival of dl at the enhancer site (which we will hereafter refer to as signal noise), the effective concentration of dl in nucleus *h* is calculated as $U_{eff}^{h}=U_{nuc}^{h}+\eta 𝓝(0,1)\sqrt{U_{nuc}^{h}}$, where 𝓝(0, 1) is a random number selected from the standard normal distribution and *η* is a tunable constant. (Note: we assume that random fluctuations in the *actual* concentration of nuclear dl are small compared to the fluctuations in *effective* concentration due to the random arrival of dl molecules at enhancer sites.) The value of *η* is chosen to be between 0.02 and 0.5. Values of $U_{eff}^{h}$ that dip below zero are set to zero.

### Optimization

We use a custom evolutionary algorithm, based on a (*λ* + *μ*)-EA strategy [15, 20–22], to optimize the parameters of the model to fit the dl-Venus fluorescence data [10]. The following is a brief description (for full detail, see S1 Text). We start each optimization run with *λ* = 500 “individuals” that possess random assignments for each of the 15 parameters, chosen from a discrete set of numbers ({1 2 3 4 … 9} × 10^{−2 −1 0}^). (Based on our preliminary simulations, the starting intercompartmental exchange parameters and the dl/Cact dissociation rate (*β*_0_) are divided by 100 to avoid starting the search with unrealistically high values of these parameters.) The simulation results for nucleus *h* at time index *k*, $Y_{hk}^{N}(P^{N})$, corresponding to a set of parameters *P* ^*N*^ = [*p*_1_ *p*_2_ *p*_3_…*p*_15_]^*N*^, are evaluated and compared to the data using a residual sum of squares calculation:

$$\begin{array}{c} P=\left\{\begin{array}{c} {\left[p_{1}\;p_{2}\;p_{3}\ldots \;p_{15}\right]}^{1}\\ {\left[p_{1}\;p_{2}\;p_{3}\ldots \;p_{15}\right]}^{2}\\ \vdots \;\;\;\vdots \;\;\;\vdots \;\;\;\vdots \\ {\left[p_{1}\;p_{2}\;p_{3}\ldots \;p_{15}\right]}^{\lambda } \end{array}\right. \end{array}$$ (10)

$$\begin{array}{c} Y_{hk}^{N}\left(P^{N}\right)=U_{nuc}^{h}\left(t_{k};P^{N}\right)+W_{nuc}^{h}\left(t_{k};P^{N}\right) \end{array}$$ (11)

$$\begin{array}{c} S^{N}=\frac{1}{n_{h}n_{k}}\sum_{h=1}^{n_{h}}\sum_{k=1}^{n_{k}}X_{hk}Y_{hk}^{N}/\frac{1}{n_{h}n_{k}}\sum_{h=1}^{n_{h}}\sum_{k=1}^{n_{k}}{\left(Y_{hk}^{N}\right)}^{2}=\frac{\bar{XY^{N}}}{\bar{{\left(Y^{N}\right)}^{2}}} \end{array}$$ (12)

$$\begin{array}{c} P_{RSS}^{N}=\sum_{h=1}^{n_{h}}\sum_{k=1}^{n_{k}}{\left(\left(X_{hk}-S^{N}Y_{hk}^{N}\right)/dX_{hk}\right)}^{2}, \end{array}$$ (13)

where *X*_*hk*_ = dl-Venus data, *dX*_*hk*_ = measurement uncertainty—both corresponding to simulated nucleus *h* and timepoint *k*—and *S* ^*N*^ is the ordinary least squares estimate of the scale factor that minimizes the difference between *X* and *Y* across all time and space coordinates and for parameter set *N*. *U*_*nuc*_ and *W*_*nuc*_ are the dimensionless versions of nuclear dl and nuclear dl/Cact complex, respectively (see Equations (1) and (3)). The dl-Venus data are taken directly from Reeves et al. [10], in which the concentration of nuclear Venus-tagged dl was imaged in live embryos and quantified in space and time and averaged.

The top *μ* = 100 individuals are then grouped into sets of 5 that “mate” to form “children” by producing new individuals whose parameters are random combinations of two randomly chosen “parents.” Each parameter of the “children” set is then randomly mutated with a bias toward the parameters of the top-ranked individual in the “parent” set. The 500 “children” are then run through the simulation and ranked, and the top 100 individuals from the combined pool of parents + children are used to generate the next generation, and the process repeats. We have observed this process to stagnate at about 25 generations. After the 25th generation, we keep the top 100 parameter sets as the end product of each evolutionary optimization run.

A similar method is used to find parameter sets for simulations of gene expression, with *λ* = 250, *μ* = 50. For each optimization run, we use a set of parameters that are randomly chosen from the top 10% of those collected to simulate the dl/Cact dynamics. We use the dl/Cact dynamics associated with this set of parameters as an input to the gene expression model equations, and allow only the gene expression parameters to evolve for 9 different values of the noise parameter, *η*, between 0.02 and 0.5. Since the concentration range of the simulated dl gradient is known to be between approximately 0 and 3 prior to optimization, the initial threshold (*θ*_*i*_) parameters are restricted to random numbers between 0 and 3. For simplicity, the lifetime parameters (*τ*_*i*_) are also restricted to this range, in minutes, but often evolve to values outside of that range. Again, we have observed this process to stagnate at about 25 generations. After the 25th generation, we keep the top 50 parameter sets as the end product of each evolutionary optimization run.

## Results

### Simulations of dl/Cact dynamics

To obtain an accurate picture of dl/Cact dynamics, we formulated a model of the dl gradient based on previous work ([15]; see S1 Text for details) and fit the model to a set of detailed spatiotemporal data for the dl gradient, obtained by live imaging of dl tagged with YFP variant Venus [10].

**Fig 3:**
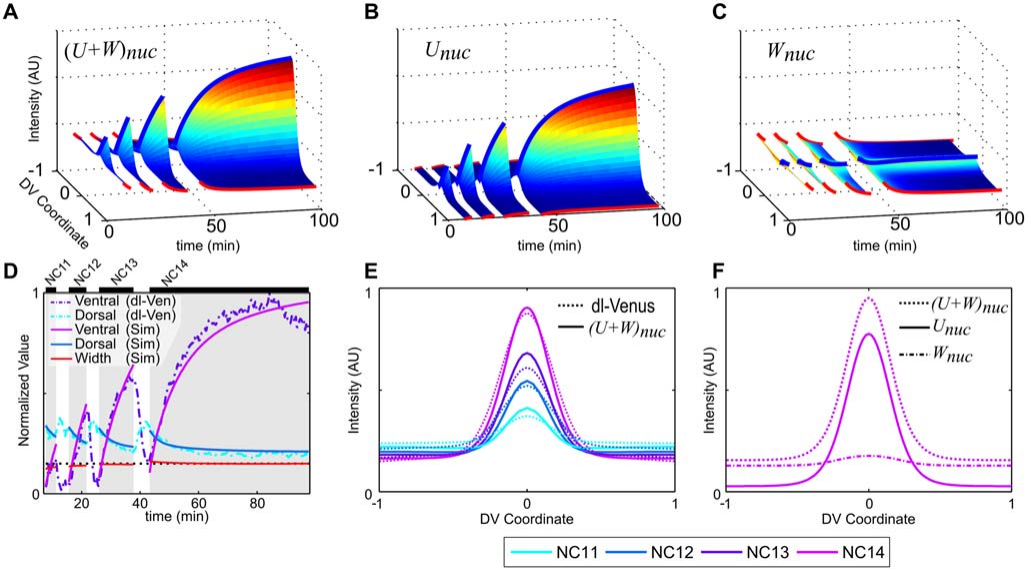
Model results with nuclear dl/Cact. (a) Surface plot of the best-fit parameter set (*U*_*nuc*_ + *W*_*nuc*_). (b) Surface plot of nuclear free dl (*U*_*nuc*_, Equation 1). (c) Surface plot of nuclear dl/Cact complex (*W*_*nuc*_, Equation 3). (d) The best-fit simulation result compared to the nuclear dl-Venus data from [10], normalized to dl-Venus maximum. Width refers to *σ* value when fit to a Gaussian function; dotted line represents average dl gradient width of 0.15 as in [10]. (e) Comparing snapshots at the end of each nuclear cycle. (f) Comparison of NC14 gradient snapshots.

Our modeling results largely agreed with previously published observations. Specifically, we were able to simulate the overall increase in nuclear dl seen along the ventral midline (Fig. 2a), and the overall spatiotemporal shape of the simulated data set was in qualitative agreement with the dl-Venus data set (Fig. 2b; compare with Fig. 3a). However, our model could not simulate the decrease in nuclear dl seen in the dorsal-most nuclei (see Fig. 2a).

Based on the dl-Venus data set, it was proposed that the dorsal-most nuclei begin each interphase with “too much” dl-Venus, as a result of nuclear dl levels being in equilibrium with the cytoplasm [10]. In the model, however, the initial conditions for each interphase include newly-formed nuclei that are devoid of any dl, Cact, or dl/Cact complex. This simplifying assumption precludes the possibility that dl/Cact complex can ever be found in the nuclei. Therefore, we formulated an “extended model” in which we removed this simplifying assumption but left the kinetic interactions the same. Specifically, our new model assumed that as nuclear envelopes reform in the cytoplasm, the contents of the nucleus would simply reflect that of the cytoplasm at the start of interphase (a straightforward assumption, given the porosity of nuclear envelopes during mitosis [23, 24]). This entailed modeling not only nuclear dl, but also nuclear Cact and nuclear dl/Cact complex, as each nascent nuclear compartment envelops all cytoplasmic species at the beginning of interphase. In other words, even if the nuclear import rates of Cact and dl/Cact complex were zero, the initial conditions for each interphase would include non-zero levels of these species in the nuclei.

If dl/Cact complex does indeed reside in the nucleus to some degree, it seems reasonable to assume this molecular species also contributes to the nuclear dl-Venus fluorescence. If dl/Cact complex contributes to fluorescence and not gene expression, this result also provides a straightforward solution to the previously-noted difference between the dl gradient’s spatial range as measured by fluorescence and its spatial range as assayed by its ability to specify target gene locations (for example, see [10]). Therefore, under this extended model, we take nuclear dl fluorescence intensity to result from the sum of contributions from free dl and dl/Cact complex (see Equation 11). We thus fit the sum of free nuclear dl (1) and nuclear dl/Cact complex (3) in our extended model to the dl-Venus data set in both space and time, revealing parameter sets that show an excellent fit to the data (see Fig. 3d). In particular, the extended model captures the decreasing basal levels, which affirms our assumption that nuclei begin interphase with the same concentration profile as the cytoplasm.

Using our best-fit results of the extended model, we can separate the experimentally-measured dl-Venus fluorescence into an inactive, dl/Cact component and an active, free nuclear dl component by considering the numerical solutions to equations (3) and (1) separately (Fig. 3a-c). Our model predicts that in the lateral and dorsal regions of the embryo, the observed levels of dl-Venus fluorescence, including the non-zero basal levels, are predominantly composed of dl/Cact complexes, and that the free nuclear dl gradient decays to near zero levels.

### Parameter analysis of extended dl/Cact model

Each evolutionary optimization run that was used to fit our model to the dl-Venus data set resulted in 100 closely-clustered parameter sets. We represent each evolutionary optimization run by an average parameter set, in which we calculate the mean and standard deviation (weighted by RSS error; see Methods and S1 Text) for each parameter, across all 100 sets. We collected 254 such runs, resulting in 254 average parameter sets that are nearly indistinguishable in terms of their average RSS error (see Methods and S1 Text), yet vary in the values of their parameters, as has been observed previously for biological models [25, 26]. Despite the variation in parameters, several trends among the parameter sets have surfaced, most notably among the nuclear import/export rates of the three molecular species.

**Fig 4:**
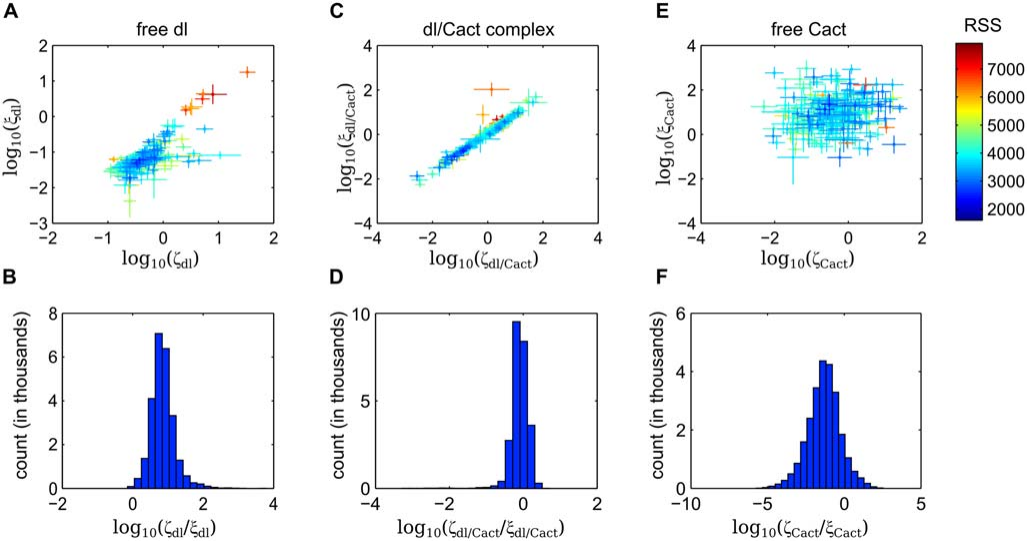
Import/export parameter analysis. (a,c,e) Scatter plot of logarithms of nuclear export vs import rates for dl (a), dl/Cact (c) and Cact (e). Each point is a weighted mean of the 100 parameter sets from a single evolutionary optimization run, with error bars representing the weighted standard deviation for those parameter sets. Color indicates weighted median RSS error for each set. (b,d,f) Equilibrium constant distributions: logarithms of quotients of all import/export parameter values (*ζ*_*i*_ and *ξ*_*i*_, respectively).

Both the import and the export rates of dl largely cluster in a small region of parameter space (Fig. 4a). Deviations from this cluster are highly correlated between these two parameters, with a sparse, high-RSS-error tail. Thus, the nuclear import equilibrium constant (the ratio of import to export) for dl is largely contained within one order of magnitude and, perhaps unsurprisingly, favors import (Fig. 4b).

On the other hand, the import and the export rates of dl/Cact complex individually appear relatively unconstrained, with both parameters spanning four orders of magnitude (Fig. 4c). However, it is clear from this plot that these two parameters are highly correlated. Indeed, their ratio, representing the nuclear import equilibrium constant for dl/Cact complex, is largely constrained to one order of magnitude, which slightly favors export (Fig. 4d).

In contrast to both of these rate constraints, the Cact import/export rates are individually relatively unconstrained, and uncorrelated (Fig. 4e,f). This is relatively unsurprising as, in our analysis, free Cact is not explicitly fit to any experimental measurements.

### Gene expression simulations

Previous work used the dl-Venus data collected in live embryos as the input to a preliminary model of gene expression [10]. While it was understood there were likely other factors involved in the expression of dl target genes, the modeling work revealed the extent to which the measured dl fluorescence alone could specify gene expression patterns. In particular, these simulations resulted in acceptable fits for Type I genes, adequate fits for Type II genes, and mediocre fits for Type III genes (which have dl-dependent boundaries past 40% DV axis; [10, 13]). The mediocre fits for the Type III genes are due to the measured spatial range of the dl nuclear fluorescence gradient being too narrow to account for the Type III genes. In light of our modeling work here, the other factors involved in DV gene regulation may include modulation of the dl nuclear gradient by nuclear Cact. Indeed, the difference in using fluorescence as a direct measurement of active dl (vs. total dl) becomes greater further away from the ventral midline, so that Type III genes such as *sog* and *zen* are the most sensitive to this interpretation of the data. Therefore, in order to investigate how our proposed interpretation of the dl fluorescence data could impact our understanding of dl-dependent patterning, we built a model of gene expression.

We began with the numerical solution for free nuclear dl (*U*_*nuc*_, Equation 1) as the input to the gene expression model. To account for the error in gradient read-out inherent to the stochastic process of dl arriving at the enhancer site, we added noise to the concentration of dl to calculate an effective dl concentration seen by each gene’s enhancer. For each nucleus *h*, we calculated $U_{eff}^{h}=U_{nuc}^{h}+\eta 𝓝(0,1)\sqrt{U_{nuc}^{h}}$, where 𝓝(0, 1) is a random number selected from the standard normal distribution and *η* is a tunable “noise” constant. A separate amount of noise was added for the calculation of each gene.

**Fig 5:**
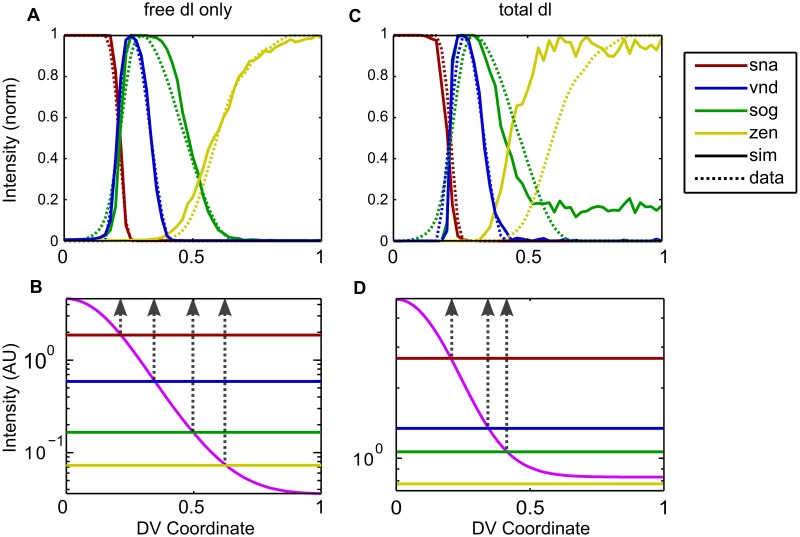
Gene expression simulations. (a, b) Using the free (active) dl gradient, we were able to accurately simulate the gene expression patterns measured with FISH [10]. (c, d) Using the total dl gradient, we obtain poor fits to Type III genes (*sog*, *zen*), as was expected. In (b, d) the fuchsia curve indicates the active dl gradient at the end of NC14, plotted on a log scale. Horizontal lines indicate median threshold parameter values, and vertical dotted lines indicate where in the DV axis we expect the final expression borders of each gene, according to where the gradient crosses the threshold. Note that the *zen* threshold is below the NC14 gradient in the total dl case (c, d), and that *zen* expression is thus a result of noise. (Note: each run is an average of 10 runs for each parameter set to reduce randomness in the plot due to noise.)

Using evolutionary optimization, we found parameter sets that show excellent agreement with the fluorescence *in situ* hybridization (FISH) data for *sna*, *vnd*, *sog*, and *zen* (Fig. 5a). In particular, our results indicate that the dl nuclear gradient can indeed pattern Type III genes, perhaps due to the fact that the nuclear concentration of free (active) dl drops by another order of magnitude past 40% DV axis (Fig. 5b), in constrast to total dl, which drops by only 20% in the same range (Fig. 5d). As shown in Fig. 5b, the gene expression thresholds cross the NC14 dl gradient at about the same location as the half-max of the corresponding gene’s boundary.

To compare the simulated gene expression patterns resulting from the free nuclear dl gradient with an undifferentiated model, we asked what gene expression patterns would result from using the combined, total nuclear dl gradient (*U*_*nuc*_ + *W*_*nuc*_) as an input to the gene expression model. In those simulations, we observe that Type III genes cannot be properly specified by the total dl gradient (Fig. 5c). This is the same trend as seen previously [10]. Several results arise from this formulation that show the model is highly susceptible to noise. First, the locations where the predicted gene expression thresholds cross the dl gradient in this case do not align with the putative locations of the gene expression boundaries (Fig. 5d). In other words, much of gene expression does not result from a straightforward interpretation of the gradient, but instead may rely on local fluctuations in the effective concentration. Second, the dynamic range (defined as *c*(*x*_1_)/*c*(*x*_2_), for any choice of *x*_1_ < *x*_2_) of dl gradient interpretation is very narrow, with genes of Types I, II, and III+ having thresholds all within a three-fold range (Fig. 5d). Such a low dynamic range could prevent the different cell fates from being properly established. Third, the best-fit threshold for Type III- genes is so low that the basal levels never drop below this threshold, and Type III- genes are only expressed as a result of dl gradient read error (noise). This phenomenon is also observed in the Type III+ genes, as these simulations show they are expressed all the way to the dorsal midline.

### Sensitivity analysis of gene expression model

Previous studies using total dl as the input to the gene expression model have revealed a high level of sensitivity of the Type III genes to changes in model parameters [10]. To determine the sensitivity of our results using free dl with respect to changes in model parameters, we took the best fit parameters for both free dl and total dl and varied them by ±10%. In both of these scenarios, we used *η* = 0.2.

**Fig 6:**
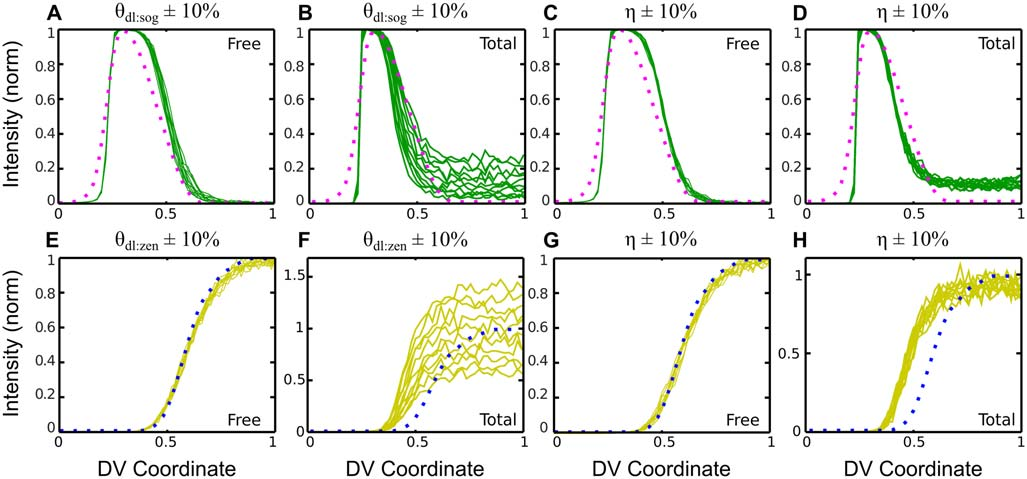
Gene expression parameter sensitivity analysis. (a-d) A 10% change in dl-sog threshold, *θ*_*dl*:*sog*_ (a,b), or noise parameter, *η* (c,d), causes a greater difference in the total dl case than the free dl only case. (e-h) In the same way, a 10% change in dl-zen threshold, *θ*_*dl*:*zen*_ (e,f), or noise parameter (g,h) causes a greater difference to the total dl case than the free dl case. For each case, sensitivity was analyzed using the best parameter set for *η* = 0.2, and each line shown is the average of 10 simulations. The data to which the model was optimized are shown as dotted lines. (Note: each run is an average of 10 runs for each parameter set to reduce randomness in the plot due to noise.)

The model with free dl is insensitive to 10% variations in the three model parameters (*θ*_*dl*:*mRNA*_, *τ*_*mRNA*_, *η*) for each gene tested (Fig. 6). In contrast, the Type III genes in the model using total dl are highly sensitive to the value of *θ*_*dl*:*mRNA*_ as well as *η*. This result emphasizes that our understanding of how the dl gradient specifies genes far away from the ventral midline is contingent on the proper interpretation of dl fluorescence measurements.

In our gene expression model, we idealize the relationship between dl concentration and gene expression rate as a hard-threshold (Hill coefficient *n*_*H*_ = 100). Even with such a strict phenomenology, our extended model can reproduce the non-sharp boundaries of gene expression, as exhibited by the Type III genes and, to a lesser extent, the Type II genes [10]. Specifically, our results indicate that the graded nature of the boundaries of each gene may be a direct result of the amount of gradient read error (noise), which confirms previous results based on using fluorescence directly as a measure of active dl [10]. However, if we model gene expression using a shallower Hill function (*n*_*H*_ = 4), our results are essentially unchanged (S4 Fig.).

### Background subtraction mechanism allows the dl gradient to overcome noise

**Fig 7:**
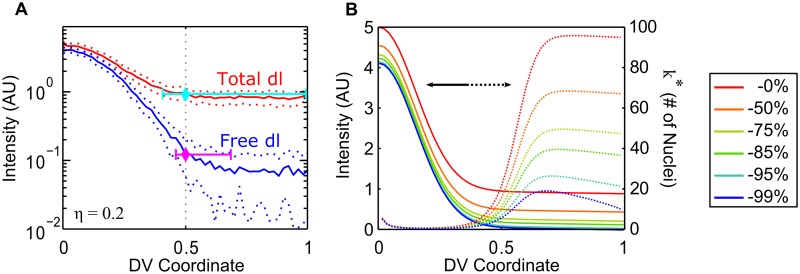
Effect of Cact background subtraction. (a) From raw fluorescence measurements (red), lateral and dorsal nuclei should have trouble interpreting their position in the DV axis because of signal noise (dotted curves). If only the active pool of dl is taken into account (blue), noise is not prohibitive to accurate boundary placement, meaning that dl can indeed pattern the whole of the DV axis. (b) If we consider a simple example of background subtraction, the shape of the dl gradient remains the same (solid lines, from red to blue) while the relative error due to noise (dotted lines, in no. of nuclei) at each point in the DV axis significantly decreases (especially beyond 40% DV) as a greater percentage of the basal concentration is subtracted.

As seen from our dl gradient and gene expression simulations (Fig. 5), subtracting the inactive component of the dl gradient from that measured by fluorescence reveals an active dl gradient that is able to convey spatial information over a greater proportion of the DV axis due to its expanded dynamic range. One may ask how this “background subtraction” mechanism achieves this. As illustrated in Fig. 7a, background subtraction via Cact increases the relative difference in active dl concentration between the ventral and dorsal midlines. This effect is achieved even if the dl/Cact profile is flat, in which case the shape and slope of the dl activity gradient is the same as that of the total dl gradient, but the dynamic range is greatly improved. According to our analysis, this becomes most important in the issue of overcoming noise. To quantify this effect, we calculate the potential for nuclei to misinterpret their position in the *x*-direction due to noise (Fig. 7b).

Consider two nuclei that are separated as *k*-nearest neighbors, so that they are Δ*x* = *kD*_*n*_ apart, where *D*_*n*_ is the distance between nearest-neighbor nuclei. Suppose these nuclei are in a concentration gradient *c*(*x*), and a background subtracted gradient *c*_*b*_(*x*) = *c*(*x*) − *B*, where *B* is a constant less than min{*c*(*x*)}. The relative difference in concentration between the nuclei is $\frac{\Delta c}{c}\approx \frac{1}{c}|\frac{dc}{dx}|\Delta x=\frac{1}{c}|\frac{dc}{dx}|kD_{n}$. In the same general region of the embryo, the relative amount of noise is $\frac{\delta c}{c}=\frac{\eta }{\sqrt{c}}$. In order for these two nuclei to perceive different enough dl concentrations to overcome the noise, one must have $\frac{\Delta c}{c}\geq \frac{\delta c}{c}$, or $k\geq \frac{\eta \sqrt{c}}{|\frac{dc}{dx}|D_{n}}$. However, for the background subtracted gradient, the constraint is $k\geq \frac{\eta \sqrt{c-B}}{|\frac{dc}{dx}|D_{n}}$, which is much easier to overcome.

In other words, for nuclei separated as $k^{*}=\frac{\eta \sqrt{c}}{|\frac{dc}{dx}|D_{n}}$ nearest neighbors, the lower the value of *c*, the smaller k* need be, as long as the slope remains constant. This effect is plotted in Fig. 7b, in which the solid lines are a representative dl gradient with more and more of the basal levels subtracted uniformly (red to blue), and the dotted lines represent the corresponding k*, the number of nuclei misinterpreting their position along the axis. Past 40% DV position, the value of k* is dramatically smaller with a larger background subtraction. Therefore, nuclei patterned by a “background subtracted” dl gradient, one in which the nuclear dl levels are closer to zero (but the slopes are the same), will be better able to distinguish themselves from their closely-separated neighbors.

## Discussion

**Fig 8:**
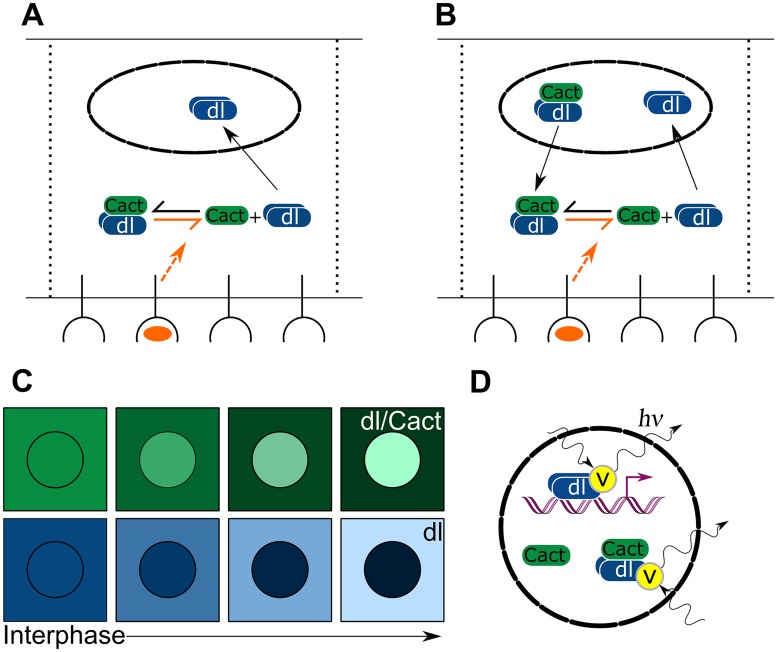
Summary. (a) In the classical model of dl/Cact dynamics, the import of dl is of primary concern. (b) In our extended model, the coexistence of dl, Cact and dl/Cact complex due to nuclear porosity must be taken into account to interpret the results of fluorescence studies. (c) Our modeling results predict the export of dl/Cact complex from dorsal nuclei contributes to a decrease in nuclear fluorescence, and the import of cytoplasmic dl contributes to the increase in nuclear fluorescence in ventral nuclei. (d) In all nuclei, free dl and dl/Cact complex are both fluorescently tagged but only free dl contributes to gene expression.

Recently, detailed measurements of morphogen gradients have led to rapid advances in our understanding of morphogen dynamics. However, questions remain as to how the spatial information carried by a morphogen gradient results in gene expression boundaries. In this study, we attempt to address this question by analyzing a mechanistic model of dl/Cact dynamics in the early embryo in light of recent live imaging measurements of nuclear dl-Venus fluorescence [10]. In comparing our modeling results against the experimental data, we found that only when our model includes nuclear Cact and nuclear dl/Cact complex can it account for experimental observations such as the declining basal levels of dl-Venus fluorescence (Fig. 8a-c). In such a model, the most straightforward assumption is that fluorescence measurements are a combination of both free nuclear dl and dl/Cact complex (Fig. 8d). This assumption implies that the dl nuclear concentration gradient, as measured by fluorescence, represents total nuclear dl, and not necessarily the gradient of dl activity. This is especially true on the lateral and dorsal side of the embryo, where dl/Cact complex becomes the dominant dl-containing species.

Our separation of the dl-Venus data into active and inactive pools of nuclear dl suggests a solution to the question of whether the observed dl gradient, without separation, is too narrow to carry information past 40% DV axis (a problem of some disagreement: see [10, 12, 15, 16, 27]). When we distinguish between free nuclear dl and nuclear dl/Cact complex, the resulting active gradient possesses a larger dynamic range than when measured by fluorescence alone (see Fig. 7a), and thus is capable of generating expression patterns that accurately reflect what is measured *in vivo*. Indeed, this result is borne out in our simulations of gene expression patterns.

Our predictions of gene expression patterns using computationally separated dl fluorescence measurements are also significantly more robust to variations in both the overall noise parameter and the individual threshold parameters when compared to simulations based on raw fluorescence (Fig. 6; see also S1–S4 Figs.). Using raw fluorescence becomes especially problematic in lateral and dorsal regions of the embryo, as the difference in dl-fluorescence measurements between neighboring nuclei becomes vanishingly small, resulting in lateral and dorsal nuclei becoming almost indistinguishable in their predicted gene expression (see Fig. 7b). For example, Fig. 6b shows that when *sog* expression best fits the FISH data near 40% DV axis, the nuclei beyond 40% DV almost uniformly express *sog*, creating a “tail” of *sog* expression that does not match the data. However, raising the threshold value (*θ*_*dl*:*sog*_) in order to eliminate the “tail” results in a very poor fit in which the *sog* domain collapses to more closely resemble the *vnd* domain, as described previously [10]. In addition, our optimization results found that, in the raw fluorescence case, the threshold for *zen* repression is actually below the concentration of dl, meaning that expression of *zen* is only permitted because signal noise in the dorsal half of the embryo causes the effective concentration of dl to occasionally dip below the *zen* threshold.

In our simulations of gene expression, the added noise is consistent with realistic estimates. As speculated recently, this noise, plus a noise-filtering mechanism, may be the mechanism by which we get graded gene expression boundaries, even if the input-output response phenomenology is infinitely sharp [10]. This result is consistent with the observation that gene expression boundaries become more graded with increasing distance from the ventral midline, which is precisely what would be expected were noise to play a part in the boundary shape.

Our gene expression model did not take into account other factors that could regulate gene expression, such as Twist and Zelda [28, 29]. Including such factors would be beneficial in quantitatively understanding gene expression patterns, including timing and sharpness [30–32]. However, we do not anticipate these factors influencing our conclusion regarding the regulation of Type III genes, such as *sog* and *zen*, as Twist is a short-ranged transcriptional partner of dl [33], and Zelda expression is spatially uniform [34]. On the other hand, including Zelda in a gene expression model may help explain how a changing dl gradient can result in early gene expression [31].

Precise spatial measurements of Cact localization in the embryo have been difficult to obtain [35–37]. However, our modeling work demonstrates that the presence of Cact in the nucleus is a straightforward conclusion from the observational data. This implies Cact may have a previously unappreciated function in the nucleus to regulate the effective concentration of (transcriptionally-active) dl. In mammalian systems, I*κ*B does indeed enter the nucleus to regulate NF-*κ*B molecules [38–41]. In those cases, it is newly-synthesized I*κ*B, expressed as a result of NF-*κ*B activation, that appears to regulate nuclear NF-*κ*B. Circumstantial evidence suggests the *cact* locus may be zygotically regulated by dl activity, as binding sites for Twist, a transcriptional partner of dl, are functional within the *cact* locus [42, 43].

Several experimental methods could be used to test the conclusions of our modeling study. First, experiments could be performed to directly detect Cact within nuclei, such as by Western blot of nuclear extracts or fluorescence correlation spectroscopy. Second, a careful measurement of dl nuclear fluorescence in mutants with uniform levels of dl (and thus, uniform gene expression) would allow us to directly correlate dl nuclear fluorescence levels to gene expression. These measurements could be compared to those in wild type embryos to infer whether dl/Cact complex is a major contributor to dl fluorescence.

The question arises as to whether fluorescence measurements of morphogen gradients are generally reliable readouts of signaling activity. Such issues can be seen in other systems, notably in Dpp signaling in the *Drosophila* larval wing disc [44–46]. As a recent example, work in the Bicoid system has revealed a co-factor for Bicoid, called Dampened, that potentiates its activity [47]. Given the results of the present study, it is possible that Bicoid fluorescence readings do not accurately reflect its signaling gradient, similar to the case for dl presented here. Further experimental data is needed to verify the predictions made by our model; however, the modeling work presented here suggests that, in some cases, an accurate mathematical framework may be necessary to properly interpret fluorescence-based data.

## Supporting Information

S1 Text Detailed description of model formulation. (PDF)

S1 Fig Effect of noise on gene expression. (Left to right) Increasing the nosie parameter, *η* from 0 to 1 shows that the slopes of the gene expression boundaries approach infinity at *η* = 0, and become very noise above *η* = 0.2. (Note: each run is an average of 10 runs for each parameter adjustment to reduce randomness in the plot due to noise. This comports with the experimental data, which are the average of 10+ embryos. The same is true for S2–S4 Figs.)
(TIF)

S2 Fig Sensitivity analysis, free dl case. Using free dl as the input to the gene expression model, a sensitivity analysis shows little sensitivity to changes in the dl threshold parameters (*θ*_*dl*:*mRNA*_), lifetime parameters (*τ*_*i*_), and noise parameter (*η*) for our genes of interest. (Hill coefficient *n*_*H*_ = 100.)
(TIF)

S3 Fig Sensitivity analysis, total dl case. Using both free dl and dl/Cact complex as the input to the gene expression model, a sensitivity analysis shows high sensitivity to changes in the dl threshold parameters (*θ*_*dl*:*mRNA*_) for both Type III genes (*sog* and *zen*; green and yellow, respectively), and little sensitivity to changes in lifetime and noise parameters. (Hill coefficient *n*_*H*_ = 100).
(TIF)

S4 Fig Sensitivity analysis, free dl case, soft threshold. Using a soft threshold (*n*_*H*_ = 4) does not change the conclusions of our sensitivity analysis.
(TIF)
