# Supplementary material for: The Presence of Nuclear Cactus in the Early Drosophila Embryo May Extend the Dynamic Range of the Dorsal Gradient
Source: PLoS Comput Biol. 2015 Apr 16;11(4):e1004159. doi: 10.1371/journal.pcbi.1004159 (PMC4400154; doi:10.1371/journal.pcbi.1004159)
Supplement: S1 Text — (PDF) [file pcbi.1004159.s001.pdf]

Supporting Information for *The Presence of Nuclear Cactus in the Early* Drosophila *Embryo May Extend the Dynamic Range of the Dorsal Gradient*

Michael D. O'Connell[1], Gregory T. Reeves[1]

[1]North Carolina State University Department of Chemical and Biomolecular Engineering, Raleigh NC.

S1 Text. Detailed description of model formulation

Original model formulation

$$\frac{d\left(V_n C^h_{dl,n}\right)}{dT} = A_n\left(k_{in}C^h_{dl,c} - k_{out}C^h_{dl,n}\right) \quad (1)$$

$$\frac{d\left(V_C C^h_{dl,c}\right)}{dT} = A_m\Gamma_{dl}\left(C^{h-1}_{dl,c} - 2C^h_{dl,c} + C^{h+1}_{dl,c}\right) + V_C\left(k_D C^h_{dc,c} - k_b C^h_{dl,c}C^h_{Cact,c}\right) - A_n\left(k_{in}C^h_{dl,c} - k_{out}C^h_{dl,n}\right) \quad (2)$$

$$\frac{d\left(V_C C^h_{dc,c}\right)}{dT} = A_m\Gamma_{dc}\left(C^{h-1}_{dc,c} - 2C^h_{dc,c} + C^{h+1}_{dc,c}\right) - V_C\left(k_D C^h_{dc,c} - k_b C^h_{dl,c}C^h_{Cact,c}\right) \quad (3)$$

$$\frac{d\left(V_C C^h_{Cact,c}\right)}{dT} = A_m\Gamma_{Cact}\left(C^{h-1}_{Cact,c} - 2C^h_{Cact,c} + C^{h+1}_{Cact,c}\right) + V_C\left(k_D C^h_{dc,c} - k_b C^h_{dl,c}C^h_{Cact,c} - k_{Deg}C^h_{Cact,c}\right) + P_{Cact} \quad (4)$$

where $k_D = Aexp\left(-\frac{1}{2}\left(\frac{x}{\phi}\right)^2\right) + B$.

The original model formulation features four equations representing nuclear dl, cytoplasmic dl, cytoplasmic dl/Cact complex and cytoplasmic Cact (Eqns 1-4, respectively). In these equations, C^h_s is the concentration of species s within nuclear/cytoplasmic compartment h, where $s = \{dl, n;\ dl, c;\ dc, c;\ Cact, c\}$.

The three cytoplasmic species are exchanged between neighboring compartments at a rate Γ_s in proportion to the surface area of the two touching faces, A_m (transport in the anterior-posterior direction is ignored, as the dl gradient is assumed to be approximately unchanging along this direction). Free dl is exchanged between the nucleus and cytoplasm at rates k_{out} & k_{in} in proportion to the surface area of each nucleus, A_n. Cytoplasmic dl and Cact bind to form dl/Cact complex at a rate k_b, and the unbinding rate of dl/Cact

complex is B. Toll-mediated degradation of dl/Cact complex is goverened by a Gaussian function with parameters A and ϕ. Finally, Cact is produced uniformly at a rate P_{Cact} and degraded at a rate k_{Deg}.

In this model formulation, the nuclei are assumed well-mixed, and are initially empty at the start of each interphase. During interphase, the system is goverened by all four equations. At the end of interphase, the nuclei dissolve and nuclear dl is mixed with cytoplasmic dl within each compartment. During mitosis, the nuclei are undefined and the system is goverened by the three cytoplasmic equations, minus the terms for nuclear import/export. At the end of mitosis, the number of nuclei/compartments increases instantly, and the total concentration of each protein is interpolated into the new compartments. The concentration of nuclear dl starts at zero once again, and the next interphase begins. (See also [1].)

Updated Model Formulation

To allow for the presence of dl protein within nuclei at the start of interphase, we updated the model by removing the assumption that nuclei begin interphase empty. This updated model formulation is governed by six differential equations, now including equations for nuclear dl/Cact complex and nuclear Cact. At the beginning of each interphase, nuclear envelopes encapsulate a volume of cytoplasm as they reform. The concentration of each species is thus the same between the nucleus and cytoplasm before nuclear import/export dynamics take over.

The updated model consists of the following six differential equations:

$$\frac{d\left(V_n C^h_{dl,n}\right)}{dT} = A_n\left(k_{in,dl}C^h_{dl,c} - k_{out,dl}C^h_{dl,n}\right) - V_n\left(k_b C^h_{dl,n}C^h_{Cact,n} - k_{D0}C^h_{dc,n}\right) \tag{5}$$

$$\begin{aligned}\frac{d\left(V_C C^h_{dl,c}\right)}{dT} = {} & A_m\Gamma_{dl}\left(C^{h-1}_{dl,c} - 2C^h_{dl,c} + C^{h+1}_{dl,c}\right) + V_C\left(k_D C^h_{dc,c} - k_b C^h_{dl,c}C^h_{Cact,c}\right) \\ & - A_n\left(k_{in,dl}C^h_{dl,c} - k_{out,dl}C^h_{dl,n}\right)\end{aligned} \tag{6}$$

$$\frac{d\left(V_n C^h_{dc,n}\right)}{dT} = A_n\left(k_{in,dc}C^h_{dc,c} - k_{out,dc}C^h_{dc,n}\right) + V_n\left(k_b C^h_{dl,n}C^h_{Cact,n} - k_{D0}C^h_{dc,n}\right) \tag{7}$$

$$\begin{aligned}\frac{d\left(V_C C^h_{dc,c}\right)}{dT} = {} & A_m\Gamma_{dc}\left(C^{h-1}_{dc,c} - 2C^h_{dc,c} + C^{h+1}_{dc,c}\right) - V_C\left(k_D C^h_{dc,c} - k_b C^h_{dl,c}C^h_{Cact,c}\right) \\ & - A_n\left(k_{in,dc}C^h_{dc,c} - k_{out,dc}C^h_{dc,n}\right)\end{aligned} \tag{8}$$

$$\frac{d\left(V_n C^h_{Cact,n}\right)}{dT} = A_n\left(k_{in,Cact}C^h_{Cact,c} - k_{out,Cact}C^h_{Cact,n}\right) - V_n\left(k_b C^h_{dl,n}C^h_{Cact,n} - k_{D0}C^h_{dc,n}\right) \tag{9}$$

$$\begin{aligned}\frac{d\left(V_C C^h_{Cact,c}\right)}{dT} = {} & A_m\Gamma_{Cact}\left(C^{h-1}_{Cact,c} - 2C^h_{Cact,c} + C^{h+1}_{Cact,c}\right) + V_C\left(k_D C^h_{dc,c} - k_b C^h_{dl,c}C^h_{Cact,c}\right. \\ & \left. - k_{Deg}C^h_{Cact,c}\right) - A_n\left(k_{in,Cact}C^h_{Cact,c} - k_{out,Cact}C^h_{Cact,n}\right) + P_{Cact}\end{aligned} \tag{10}$$

where $k_D = k_D^{max} exp\left(-\frac{1}{2}\left(\frac{x}{\phi}\right)^2\right) + k_{D0}$.

Non-dimensionalizing, we have:

$$\frac{d\left(\widetilde{V}_n U_{nuc}^h\right)}{dt} = \widetilde{A}_n\left(\zeta_{dl}U_{cyt}^h - \xi_{dl}U_{nuc}^h\right) - \widetilde{V}_n\left(\gamma U_{nuc}^h Z_{nuc}^h - \beta_0 W_{nuc}^h\right) \tag{11}$$

$$\frac{d\left(\widetilde{V}_C U_{cyt}^h\right)}{dt} = \widetilde{A}_m\lambda_{dl}\left(U_{cyt}^{h-1} - 2U_{cyt}^h + C_{cyt}^{h+1}\right) + \widetilde{V}_C\left(\hat{\beta}W_{cyt}^h - \gamma U_{cyt}^h Z_{cyt}^h\right) - \widetilde{A}_n\left(\zeta_{dl}U_{cyt}^h - \xi_{dl}U_{nuc}^h\right) \tag{12}$$

$$\frac{d\left(\widetilde{V}_n W_{nuc}^h\right)}{dt} = \widetilde{A}_n\left(\zeta_{dc}W_{cyt}^h - \xi_{dc}W_{nuc}^h\right) + \widetilde{V}_n\left(\gamma U_{nuc}^h Z_{nuc}^h - \beta_0 W_{nuc}^h\right) \tag{13}$$

$$\frac{d\left(\widetilde{V}_C W_{cyt}^h\right)}{dt} = \widetilde{A}_m\lambda_{dc}\left(W_{cyt}^{h-1} - 2W_{cyt}^h + W_{cyt}^{h+1}\right) - \widetilde{V}_C\left(\hat{\beta}W_{cyt}^h - \gamma U_{cyt}^h Z_{cyt}^h\right) - \widetilde{A}_n\left(\zeta_{dc}W_{cyt} - \xi_{dc}W_{nuc}\right) \tag{14}$$

$$\frac{d\left(\widetilde{V}_n Z_{nuc}^h\right)}{dt} = \widetilde{A}_n\left(\zeta_{cact}Z_{cyt}^h - \xi_{cact}Z_{nuc}^h\right) - \widetilde{V}_n\psi\left(\gamma U_{nuc}^h Z_{nuc}^h - \beta_0 W_{nuc}^h\right) \tag{15}$$

$$\frac{d\left(\widetilde{V}_C Z_{cyt}^h\right)}{dt} = \widetilde{A}_m\lambda_{Cact}\left(Z_{cyt}^{h-1} - 2Z_{cyt}^h + Z_{cyt}^{h+1}\right) + \widetilde{V}_C\left(\psi\hat{\beta}W_{cyt}^h - \psi\gamma U_{cyt}^h Z_{cyt}^h - \alpha Z_{cyt}^h\right) - \widetilde{A}_n\left(\zeta_{cact}Z_{cyt}^h - \xi_{cact}Z_{nuc}^h\right) + 1 \tag{16}$$

where $\hat{\beta} = \beta exp\left(-\frac{1}{2}\left(\frac{z}{\phi'}\right)^2\right) + \beta_0$ and

$$\zeta_s = \frac{A_n^{14}k_{in,s}}{V_n^{14}}\bar{T} \quad \xi_s = \frac{A_n^{14}k_{out,s}}{V_n^{14}}\bar{T} \quad \lambda_s = \frac{\Gamma_s A_n^{14}}{V_n^{14}}\bar{T} \quad \gamma = k_b C_{cact}^0\bar{T} \quad \alpha = k_{Deg}\bar{T} \quad \beta = k_D^{max}\bar{T} \quad \beta_0 = k_{D0}\bar{T}$$

$$U_{nuc} = \frac{C_{dl,n}}{C_{dl}^0} \quad U_{cyt} = \frac{C_{dl,c}}{C_{dl}^0} \quad W_{nuc} = \frac{C_{dc,n}}{C_{dl}^0} \quad W_{cyt} = \frac{C_{dc,c}}{C_{dl}^0} \quad Z_{nuc} = \frac{C_{Cact,n}}{C_{Cact}^0} \quad Z_{cyt} = \frac{C_{Cact,c}}{C_{Cact}^0}$$

$$\widetilde{A}_n = \frac{A_n}{A_n^{14}} \quad \widetilde{A}_m = \frac{A_m}{A_n^{14}} \quad \widetilde{V}_n = \frac{V_n}{V_n^{14}} \quad \widetilde{V}_C = \frac{V_C}{V_n^{14}} \quad t = \frac{T}{\bar{T}} \quad \bar{T} = 1\ min \quad z = \frac{x}{L} \quad \phi' = \frac{\phi}{L} \quad \psi = \frac{C_{dl}^0}{C_{Cact}^0}$$

for $s = \{dl, n;\ dl, c;\ dc, n;\ dc, c;\ Cact, n;\ Cact, c\}$.

The gene expression model equations take the general form:

$$\frac{d}{dt}[mRNA]_i^h = \frac{1}{\tau_i}\left(f_i\left(U_{nuc}^h, [sna]^h\right) - [mRNA]_i^h\right) \tag{17}$$

where f_i is defined for each species i as the product of the appropriate on/off terms. To avoid discontinuities, we use a Hill function with $n_H = 100$ to approximate the Heaviside step function: $C^{n_H}/(\theta^{n_H} + C^{n_H})$ for production and $\theta^{n_H}/(\theta^{n_H} + C^{n_H})$ for repression:

$$f_{sna} = \frac{\left(U_{nuc}^h\right)^{n_H}}{\left(\theta_{dl:sna}\right)^{n_H} + \left(U_{nuc}^h\right)^{n_H}},\ f_{vnd} = \frac{\left(U_{nuc}^h\right)^{n_H}}{\left(\theta_{dl:vnd}\right)^{n_H} + \left(U_{nuc}^h\right)^{n_H}}\frac{\left(\theta_{sna:vnd}\right)^{n_H}}{\left(\theta_{sna:vnd}\right)^{n_H} + [sna]^{n_H}},$$

$$f_{zen} = \frac{\left(\theta_{dl:zen}\right)^n}{\left(\theta_{dl:zen}\right)^n + \left(U_{nuc}^h\right)^n},\ f_{sog} = \frac{\left(U_{nuc}^h\right)^n}{\left(\theta_{dl:sog}\right)^n + \left(U_{nuc}^h\right)^n}\frac{\left(\theta_{sna:sog}\right)^n}{\left(\theta_{sna:sog}\right)^n + [sna]^n}.$$

To simulate noise we use $U_{eff}^h = U_{nuc}^h(t) + \eta\mathcal{N}(0,1)\sqrt{U_{nuc}^h(t)}$, where $\mathcal{N}(0,1)$ is a random number selected from the standard normal distribution. Values that fall below zero are set to zero.

Nuclear/cytoplasmic dimensions

For NCs 10-13, the volume and surface area of each nucleus are calculated assuming the nucleus is a sphere with constant radius r based on measurements in [2]. For nuclear cycle 14, the volume and surface area of each nucleus are calculated assuming the nucleus is a prolate spheroid with major and minor axes b and a, respectively, where b was approximated such that $b = 2a$. The surface area and volume parameters are normalized to the area and volume measurements of a nucleus at the end of NC14 ($A_n^{14} \approx 160\mu m^2$ and $V_n^{14} \approx 190\mu m^3$).

The volume of the cytoplasm is simply the total volume of each compartment, $V = H\,(L/n_i)^2$, where $H = 25\mu m$ represents the constant height of the simulated array of compartments, $L = 270\mu m$ is the length of the simulated array (i.e., the length of half the embryo's circumference), and n_i is the number of nuclei in nuclear cycle i.

Estimation of physical parameters determining noise levels

Derivation of η

To simulate the stochastic behavior of gene expression downstream of dl, we added artificial noise to the simulated dl nuclear concentration, δU_{nuc}, in proportion to the square root of the nuclear dl concentration, U_{nuc}, with η the proportionality constant. Here we provide a detailed derivation of η .

Berg and Purcell [3] argued that a microorganism could, at best, determine the concentration, c, of molecule X within a spherical volume of radius a with a fractional error of

$$\delta c/c \sim (1.61\, DacT)^{-1/2} \tag{18}$$

where T is the measurement timescale, and D is the diffusivity of molecule X (see also [4]). Thus, we assume that the relative fluctuations in concentration of dl at its binding site, $C_{dl,n}$, are

$$\frac{\delta C_{dl,n}}{C_{dl,n}} \approx \frac{1}{\sqrt{1.61\ DaC_{dl,n}T}}, \tag{19}$$

where D is the diffusivity of dl within the nucleus, a is the length of the DNA binding site, and T is an averaging time for the cell to measure this concentration [3, 4]. If we apply non-dimensionalization to our dl nuclear concentration by substituting $C_{dl,n} = U_{nuc}\bar{C}_{dl}$, we arrive at

$$\frac{\delta U_{nuc}}{U_{nuc}} \approx \frac{1}{\sqrt{1.61\ DaU_{nuc}\bar{C}_{dl}T}}, \tag{20}$$

or, equivalently:

$$\delta U_{nuc} = \frac{1}{\sqrt{1.61\ Da\bar{C}_{dl}T}}\sqrt{U_{nuc}} = \eta\sqrt{U_{nuc}}. \tag{21}$$

Thus, the noise levels are $\delta U_{nuc} = \eta\sqrt{U_{nuc}}$, and the proportionality constant is

$$\eta = (1.61\ Da\bar{C}_{dl}T)^{-1/2} \tag{22}$$

This is the formula employed in our gene expression simulations, in which, at each time step, a random level of Gaussian noise with a mean of zero and a standard deviation of δU_{nuc} is added to the concentration of dl in each nucleus.

We estimate the following values for the physical parameters included in the calculation of η. First, the length of the dl binding site is 10 base pairs [5], meaning $a \approx 3\ nm$. Second, it seems reasonable to

assume $\bar{C}_{dl} \approx 1-10\,\text{nM} \approx 0.6-6\,{}^{\text{molc}}/_{\mu\text{m}^3}$ based on concentration measurements of Bicoid (FlyBase: FBgn0000166) [4]. Third, D is based on fluorescence correlation spectroscopy studies of Bicoid, in which its diffusivity was found to be roughly 1-10 $\mu m^2/s$ [6]. Finally, we also argue a timescale of approximately $T \approx 1-10$ min is an appropriate averaging time for transcription during the early *Drosophila* embryo (see [7]). Therefore, using the mean value for each term in (22), we find that

$$\eta \approx \left[1.61\,\left(5.5\,\mu m^2 s^{-1}\right)(3\,\text{nm})\left(2.7\,\mu m^{-3}\right)(5.5\,\text{min})\right]^{-1/2} \approx 0.2. \tag{23}$$

While this calculation does not prove that $\eta = 0.2$, it shows that our simulations are in accord with independent estimates of noise levels.

Estimation of T

For our estimate of T, we begin with the assumption that the averaging time for the concentration of dl to be read-out by a gene locus (ie, to result in a transcription decision) is related to the time between transcriptional bursts [8, 9]. Recent work on transcriptional bursting events in the early *Drosophila* embryo suggests the timescale for bursting events is roughly 1-10 min [7, 10, 11].

References

[1] Kanodia JS, Rikhy R, Kim Y, Lund VK, DeLotto R, Lippincott-Schwartz J, et al. Dynamics of the Dorsal morphogen gradient. PNAS. 2009 Dec;106(51):21707–12.

[2] Gregor T, Wieschaus EF, McGregor AP, Bialek W, Tank DW. Stability and nuclear dynamics of the bicoid morphogen gradient. Cell. 2007 Jul;130(1):141–52.

[3] Berg HC, Purcell EM. Physics of chemoreception. J Biophys. 1977 Nov;20(2):193–219.

[4] Gregor T, Tank DW, Wieschaus EF, Bialek W. Probing the limits to positional information. Cell. 2007 Jul;130(1):153–64.

[5] Ip YT, Kraut R, Levine M, Rushlow CA. The dorsal morphogen is a sequence-specific DNA-binding protein that interacts with a long-range repression element in *Drosophila*. Cell. 1991 Jan;64(2):439–446.

[6] Abu-Arish A, Porcher A, Czerwonka A, Dostatni N, Fradin C. High mobility of bicoid captured by fluorescence correlation spectroscopy: implication for the rapid establishment of its gradient. J Biophys. 2010 Aug;99(4):L33–L35.

[7] Bothma JP, Garcia HG, Esposito E, Schlissel G, Gregor T, Levine M. Dynamic regulation of eve stripe 2 expression reveals transcriptional bursts in living *Drosophila* embryos. PNAS. 2014 Jul;.

[8] Molina N, Suter DM, Cannavo R, Zoller B, Gotic I, Naef F. Stimulus-induced modulation of transcriptional bursting in a single mammalian gene. PNAS. 2013 Dec;110(51):20563–20568.

[9] Hao N, O'Shea EK. Signal-dependent dynamics of transcription factor translocation controls gene expression. Nature Struct & Mol Bio. 2012;19(1):31–39.

[10] He F, Ren J, Wang W, Ma J. A multiscale investigation of bicoid-dependent transcriptional events in *Drosophila* embryos. PLoS One. 2011;6(4):e19122.

[11] Little SC, Tikhonov M, Gregor T. Precise developmental gene expression arises from globally stochastic transcriptional activity. Cell. 2013 Aug;154(4):789–800.

Parameter	Description
A_n	Surface area of a nucleus
A_m	Area available for intercompartmental exchange
V_n	Volume of a nucleus
V_C	Volume of a compartment
A_n^{14}	Surface area of a nucleus in NC14
V_n^{14}	Volume of a nucleus in NC14
$k_{in,j}$	Nuclear import of species j
$k_{out,j}$	Nuclear export of species j
Γ_i	Intercompartmental exchange of species i
k_b	Binding rate for dl and Cact
k_{D0}	Unbinding rate for dl/Cact complex
k_D^{max}	Maximum Toll-mediated degradation of dl/Cact complex
P_{Cact}	Production rate of Cact
k_{Deg}	Degradation rate of Cact
C_i^0	Initial concentration of species i
x	DV axis coordinate
ϕ	Width of Toll signal
L	Length of the simulated region
T	Time scale

Table S1: S1 Table. Summary of model parameters

Nuclear Cycle	10	10m	11	11m	12	12m	13	13m	14
$t(end)$ (min)	3.61	7.70	11.3	15.4	21.6	25.7	37.8	43.0	97.7
Duration (min)	3.61	4.09	3.6	3.9	6.0	4.1	12.1	5.2	54.7
n	13	13	19	19	26	26	36	36	51
$\widetilde{A}_n$	1.4	-	1.5	-	1.1	-	1.2	-	1.0
$\widetilde{A}_m$	6.5	6.5	4.4	4.4	3.2	3.2	2.3	2.3	1.7
$\widetilde{V}_n$	1.7	-	1.8	-	1.1	-	1.3	-	1.0
$\widetilde{V}_C$	56	58	25	27	13	14	6.3	7.6	2.8

Table S2: S2 Table. Dimensional quantities used in model formulation

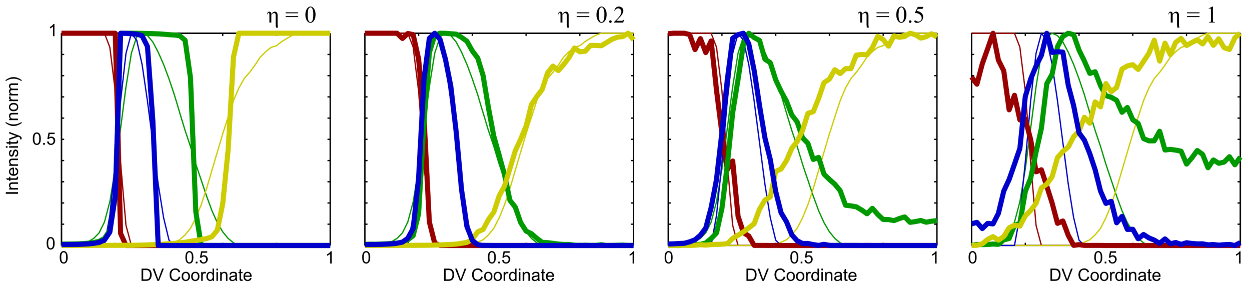

Figure S1: S1 Fig. Effect of noise on gene expression
(Left to right) Increasing the nosie parameter, η from 0 to 1 shows that the slopes of the gene expression boundaries approach infinity at $\eta = 0$, and become very noise above $\eta = 0.2$. (Note: each run is an average of 10 runs for each parameter adjustment to reduce randomness in the plot due to noise. This comports with the experimental data, which are the average of 10+ embryos. The same is true for Fig. S2-S4.)

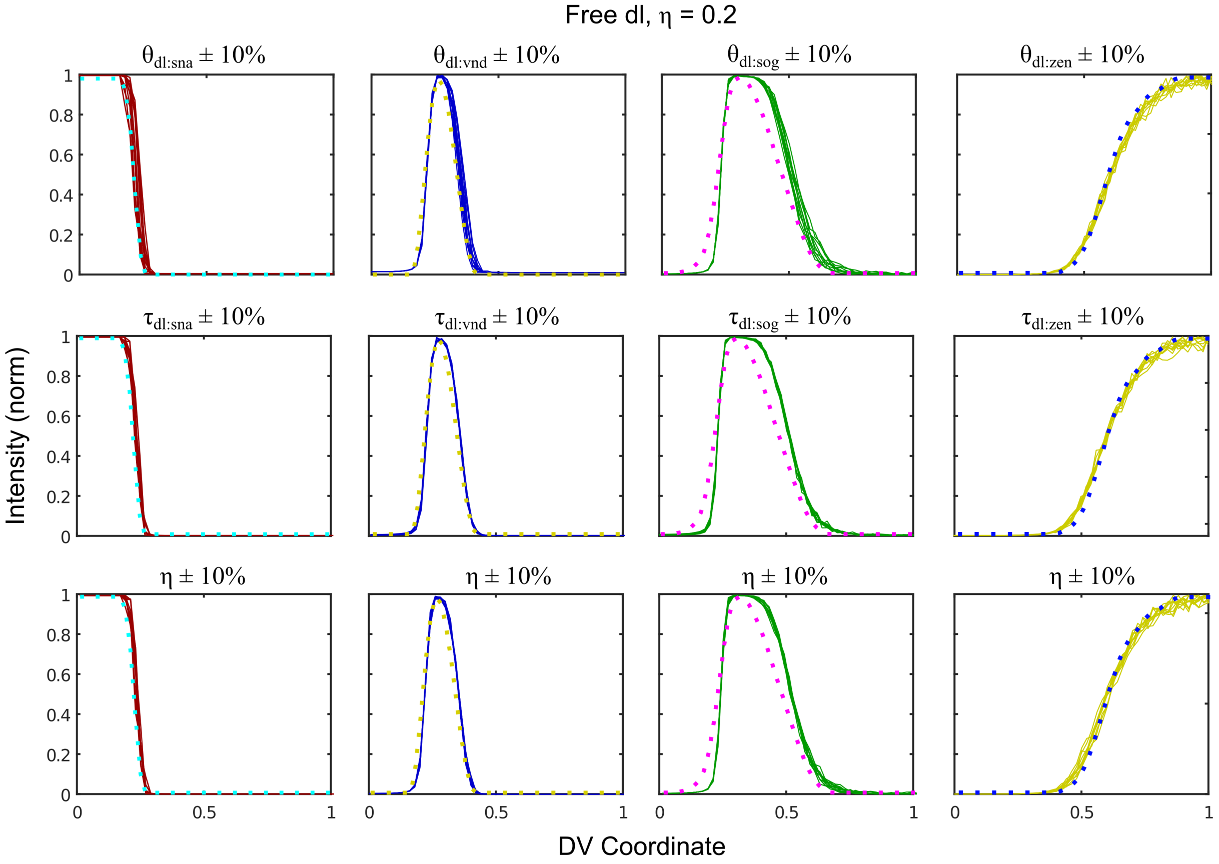

Figure S2: S2 Fig. Sensitivity analysis, free dl case

Using free dl as the input to the gene expression model, a sensitivity analysis shows little sensitivity to changes in the dl threshold parameters ($\theta_{dl:mRNA}$), lifetime parameters (τ_i), and noise parameter (η) for our genes of interest. (Hill coefficient $n_H = 100$.)

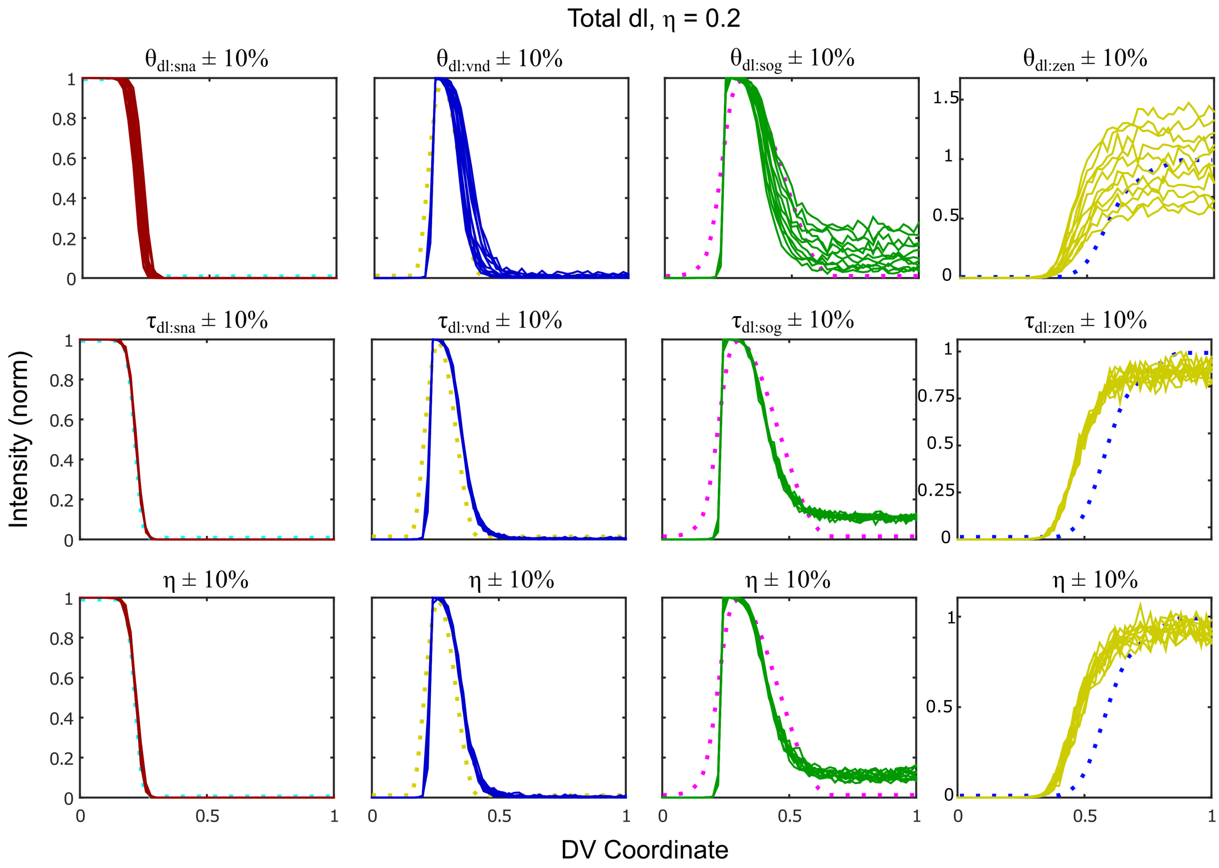

Figure S3: S3 Fig. Sensitivity analysis, total dl case

Using both free dl and dl/Cact complex as the input to the gene expression model, a sensitivity analysis shows high sensitivity to changes in the dl threshold parameters ($\theta_{dl:mRNA}$) for both Type III genes (*sog* and *zen*; green and yellow, respectively), and little sensitivity to changes in lifetime and noise parameters. (Hill coefficient $n_H = 100$).

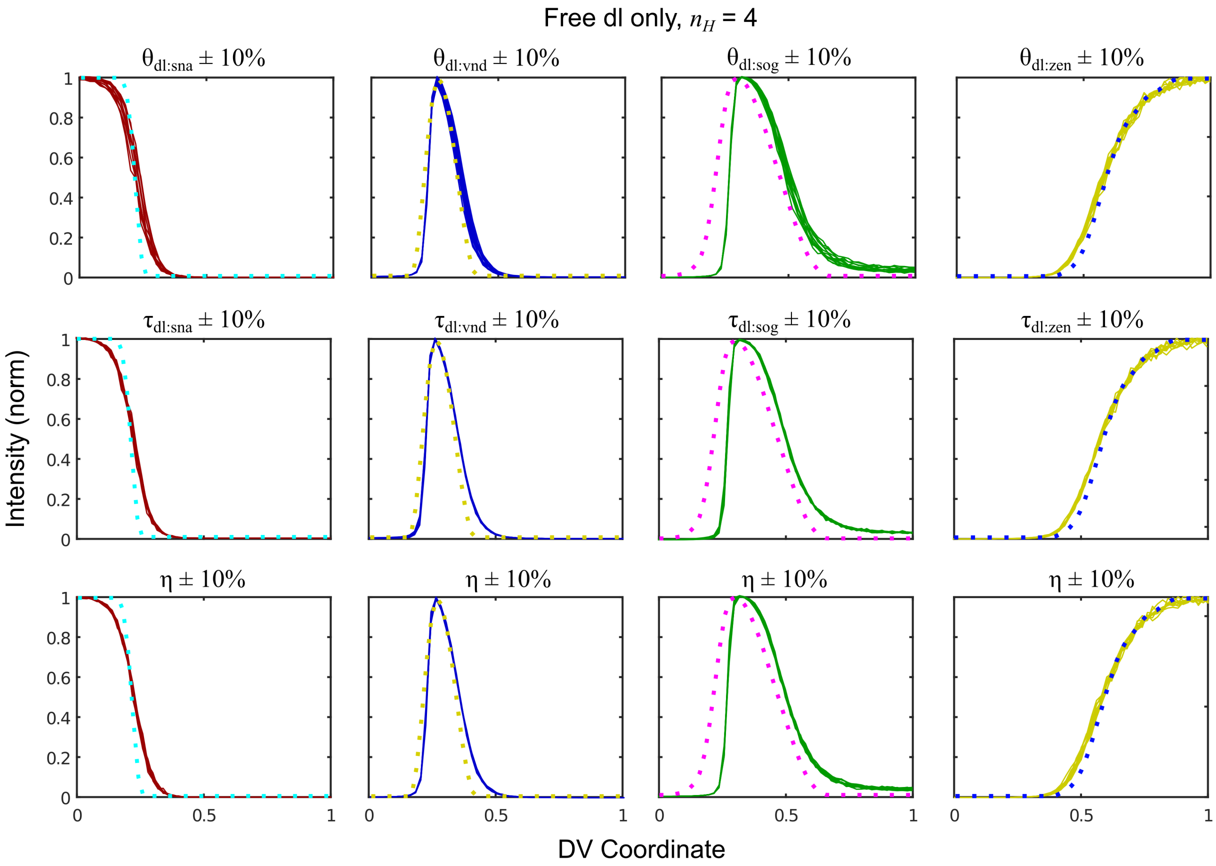

Figure S4: S4 Fig. Sensitivity analysis, free dl case, soft threshold
Using a soft threshold ($n_H = 4$) does not change the conclusions of our sensitivity analysis.
