# Supplementary figures and images for: The Presence of Nuclear Cactus in the Early Drosophila Embryo May Extend the Dynamic Range of the Dorsal Gradient
Source: PLoS Comput Biol. 2015 Apr 16;11(4):e1004159. doi: 10.1371/journal.pcbi.1004159 (PMC4400154; doi:10.1371/journal.pcbi.1004159)

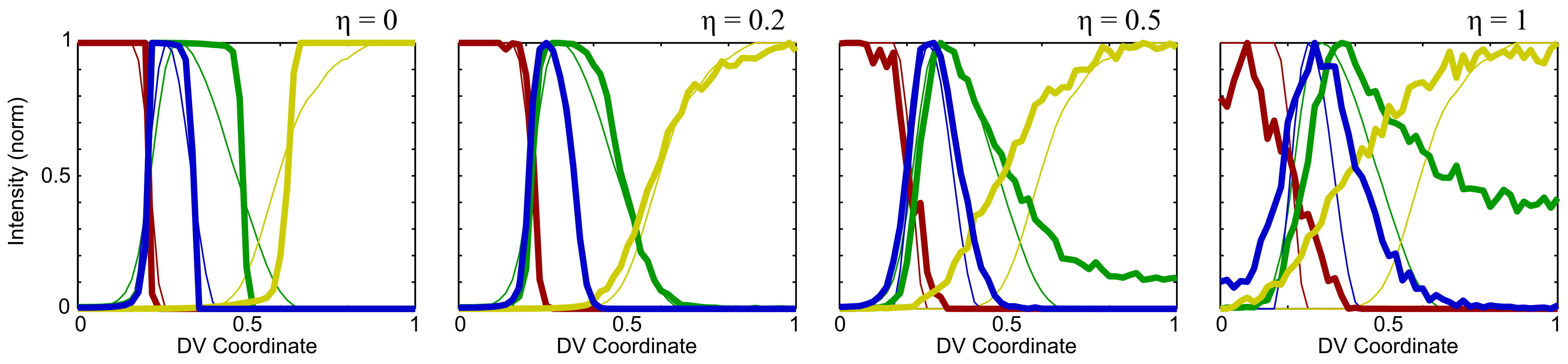

Supplement: S1 Fig — (Left to right) Increasing the nosie parameter, η from 0 to 1 shows that the slopes of the gene expression boundaries approach infinity at η = 0, and become very noise above η = 0.2. (Note: each run is an average of 10 runs for each parameter adjustment to reduce randomness in the plot due to noise. This comports with the experimental data, which are the average of 10+ embryos. The same is true for S2–S4 Figs.) (TIF) [file pcbi.1004159.s002.tif]

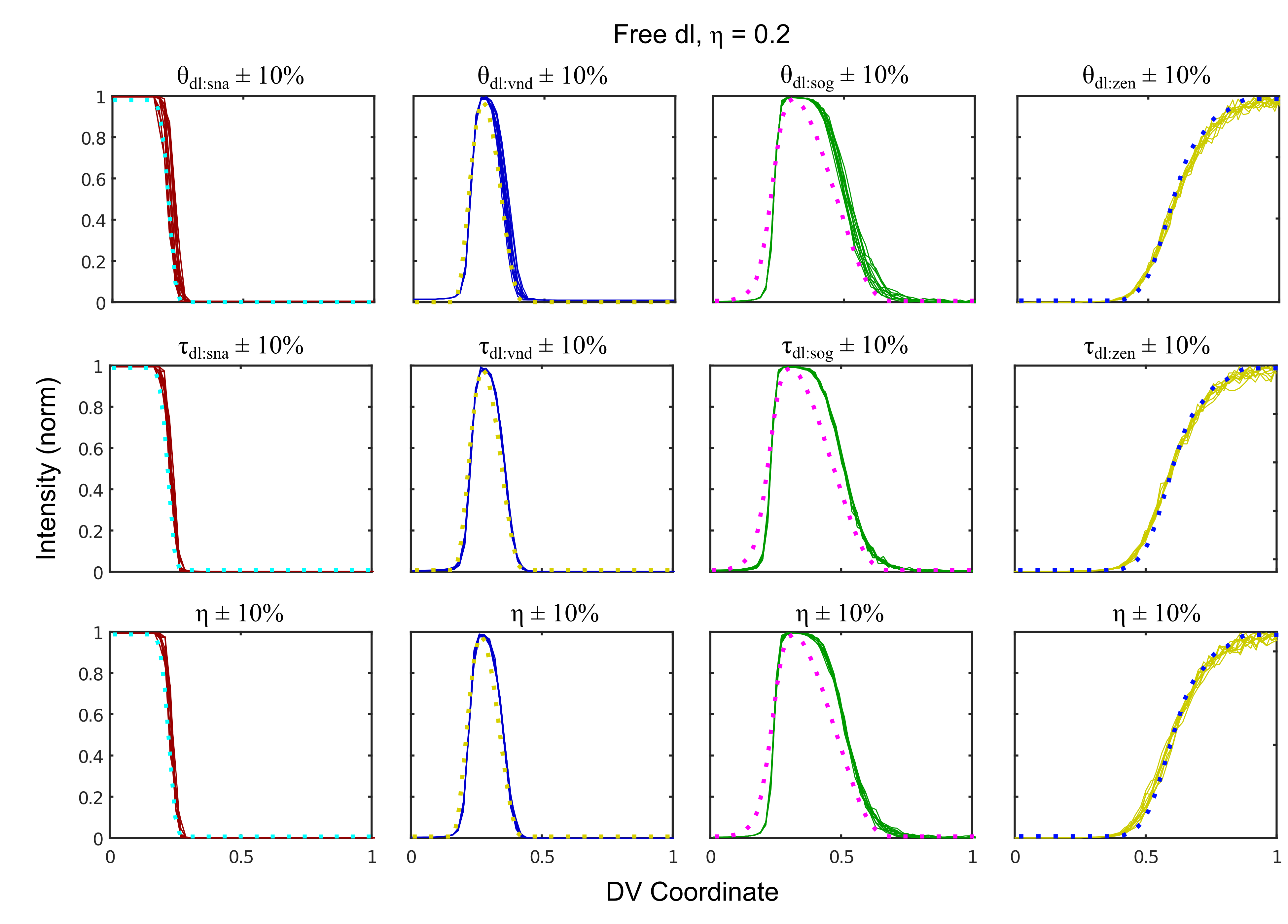

Supplement: S2 Fig — Using free dl as the input to the gene expression model, a sensitivity analysis shows little sensitivity to changes in the dl threshold parameters (θ dl:mRNA), lifetime parameters (τ i), and noise parameter (η) for our genes of interest. (Hill coefficient n H = 100.) (TIF) [file pcbi.1004159.s003.tif]

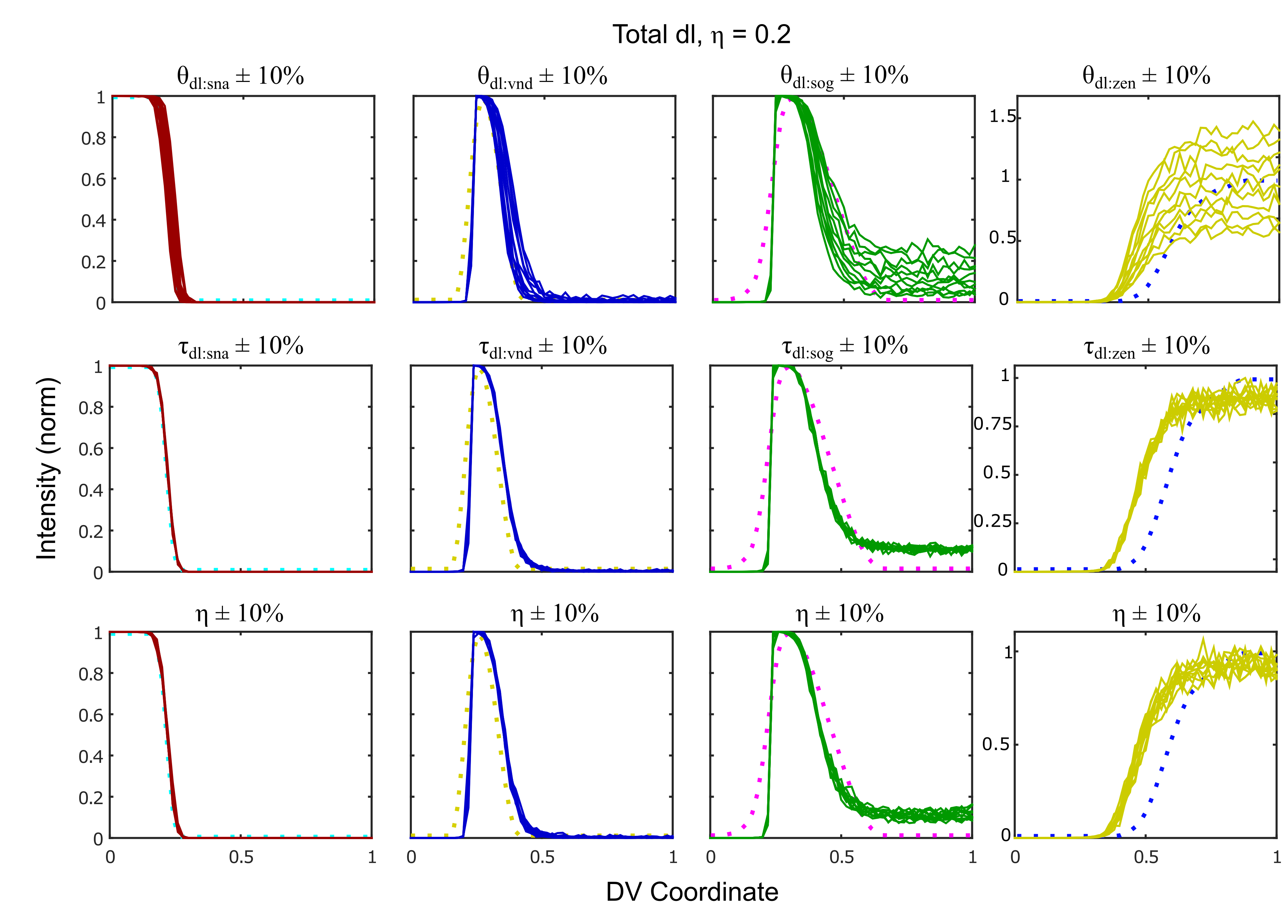

Supplement: S3 Fig — Using both free dl and dl/Cact complex as the input to the gene expression model, a sensitivity analysis shows high sensitivity to changes in the dl threshold parameters (θ dl:mRNA) for both Type III genes (sog and zen; green and yellow, respectively), and little sensitivity to changes in lifetime and noise parameters. (Hill coefficient n H = 100). (TIF) [file pcbi.1004159.s004.tif]

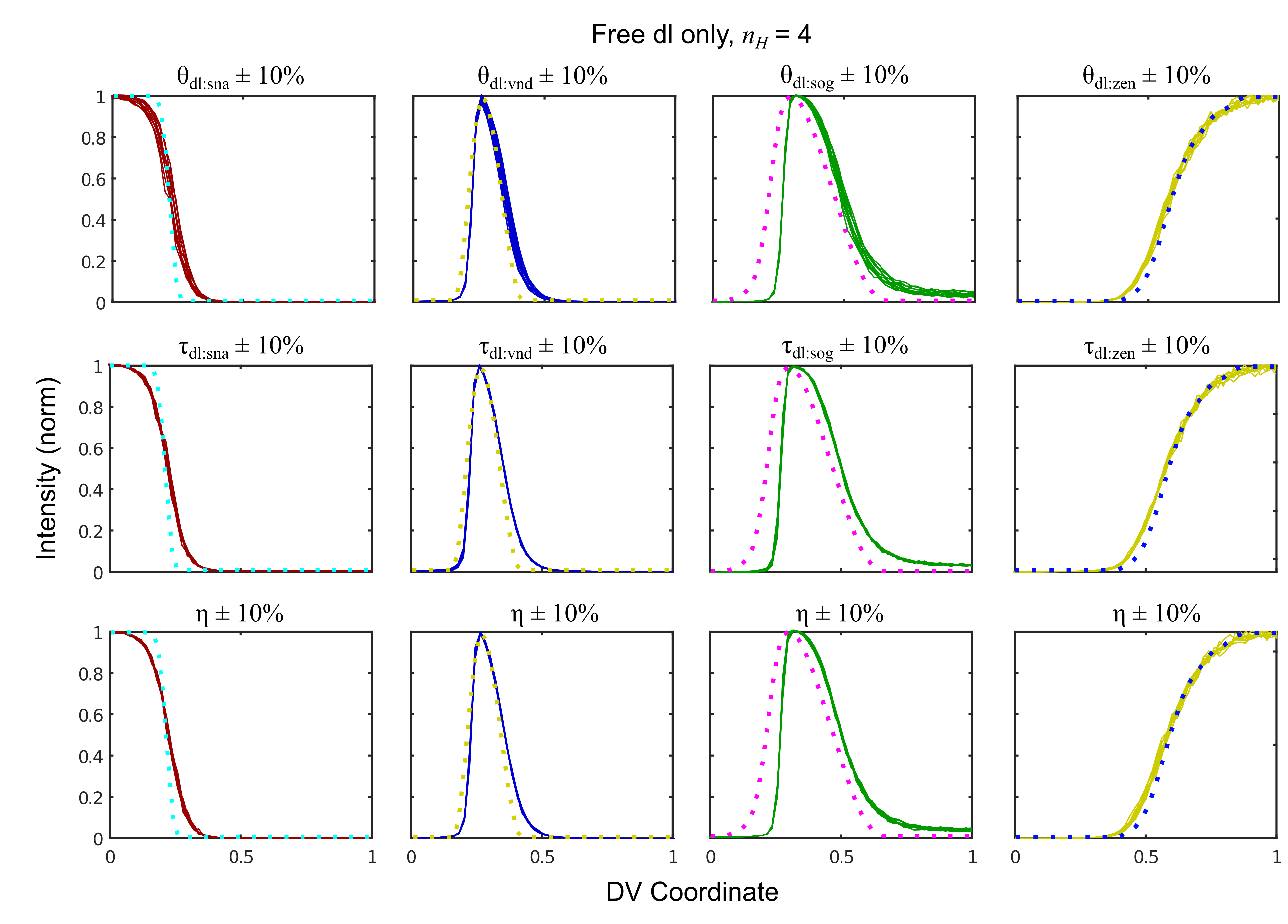

Supplement: S4 Fig — Using a soft threshold (n H = 4) does not change the conclusions of our sensitivity analysis. (TIF) [file pcbi.1004159.s005.tif]
